# Nutrient Intake and Plasma and Erythrocyte Content Among Lactating Mothers of Hospitalized Very Preterm Infants: Associations with Human Milk Composition

**DOI:** 10.3390/nu17111932

**Published:** 2025-06-04

**Authors:** Kristin Keller, Noelia Ureta-Velasco, Diana Escuder-Vieco, José C. E. Serrano, Javier Fontecha, María V. Calvo, Javier Megino-Tello, Carmen R. Pallás-Alonso, Nadia Raquel García-Lara

**Affiliations:** 1 Research Institute i+12, 12 de Octubre University Hospital, 28041 Madrid, Spain; noelia.ureta@gmail.com (N.U.-V.); diana.e.vieco@gmail.com (D.E.-V.); kpallas.hdoc@gmail.com (C.R.P.-A.); nadiaraquelg.nrgl@gmail.com (N.R.G.-L.); 2 “Aladina-MGU”—Regional Human Milk Bank, 12 de Octubre University Hospital, 28041 Madrid, Spain; 3 Department of Neonatology, 12 de Octubre University Hospital, 28041 Madrid, Spain; 4 Faculty of Medicine, Complutense University of Madrid, 28040 Madrid, Spain; 5 Department of Experimental Medicine, Faculty of Medicine, University of Lleida, 25008 Lleida, Spain; jceserrano@mex.udl.cat; 6 Food Lipid Biomarkers and Health Group, Institute of Food Science Research (CIAL), CSIC-UAM, 28049 Madrid, Spain; j.fontecha@csic.es (J.F.); mv.calvo@csic.es (M.V.C.); jamegtel@gmail.com (J.M.-T.)

**Keywords:** prematurity, diet, food consumption, nutritional circulating status, breast milk concentration, lipid profile, fatty acids, vitamins, minerals, multivariate linear regression analysis, bootstrapping

## Abstract

Background/Objectives: Lactating mothers have increased nutritional requirements, but nutritional adequacy is difficult to achieve. Additionally, human milk (HM) composition depends on maternal diet. However, the nutritional intake and status of mothers with hospitalized very preterm infants (MHVPIs) (<32 weeks of gestational age) have rarely been assessed. Hence, the aim of the present study was to determine the intake of macronutrients, micronutrients, and lipids, as well as the nutritional status of MHVPIs. The results were compared with a group of HM donors (HMDs), and associations with HM composition were evaluated using multiple linear regression. Methods: For dietary assessment, a 5-day dietary record including supplement intake was completed by 15 MHVPIs and 110 HMDs. Vitamins and fatty acids (FA) were determined in plasma and erythrocytes; minerals and methylmalonic acid were determined in urine; and macronutrients, vitamins, minerals, and the lipid profile were determined in HM. Results: Considering dietary reference intakes, the dietary evaluation of MHVPIs revealed a high percentage of inadequate nutrient intake in relation to total energy, as well as for iodine and vitamins B8, B9, C, D, and E. A high protein intake was observed. The percentage of energy from carbohydrates was low, whereas the percentage of energy from fat was high. However, the diet of MHVPIs did not differ substantially from the diet of HMDs. Associations were observed between the study groups (MHVPI vs. HMD) and the HM concentration of protein, several micronutrients, and fatty acids independent from intake and status. Conclusions: Deficient nutrient intakes did not appear to be exclusively related to MHVPI but rather seemed to be widespread in both study groups. However, for preterm infants, an insufficient supply of nutrients is critical and should be addressed in order to improve preterm infant’s outcomes. Furthermore, we provided additional insights into the exploration of HM by relating its composition to prematurity.

## 1. Introduction

An adequate diet during lactation is important for maintaining good health, not only for the mother but also for her infant, as it ensures appropriate development by providing personalized and unique nourishment, facilitating essential nutrients and biologically active components [1].

The macronutrient, micronutrient, and lipid composition of human milk (HM) depends on maternal nutrient intake, maternal nutrient stores, and nutrient production in the mammary gland [2]. Therefore, an adequate diet for the mother could lead to a better nutritional balance of diverse micronutrients in her breast milk. In this sense, several reviews have reported on the relationship between maternal dietary intake and HM composition [3,4,5,6,7]. For example, the concentration of water-soluble vitamins, as well as vitamins A and E, in HM is closely related to the maternal diet, as is the concentration of selenium, iodine, and the fatty acid (FA) profile [6,8,9,10]. The maternal nutrient status, determined by the nutrient concentration in plasma and erythrocytes rather than by body mass index (BMI), also showed relations with diverse nutrients in HM [2,3,10,11,12,13,14], such as docosahexaenoic acid (DHA), linolenic acid, linoleic acid, iron, vitamin A, and vitamin B12.

The nutritional requirements of the lactating mother are high during lactation. Hence, achieving nutritional adequacy might be difficult, as the increased demand established during gestation not only continues but also increases [1,9,15]. Even so, well-nourished mothers are generally able to produce HM containing all vitamins, minerals, and essential FAs needed by their healthy full-term infants [1,9].

However, in special circumstances, the nutritional ideality of HM might not be sufficient [16,17]. Preterm infants are born with nutritional deficits because of the early deprivation of maternal supply and the immaturity of their organs (e.g., the gut and brain, among others), which affects nutrient absorption, utilization, and retention [18]. Nevertheless, mother’s fortified own milk is the first choice of enteral feeding for preterm infants in order to meet their special nutritional needs [17,19]. However, mothers of preterm infants experience major challenges in feeding their infants and face obstacles that mothers of healthy full-term infants do not have to face [20,21].

In general, the postpartum period is a sensitive time for nutritional inadequacies [22,23,24]. Mother’s needs are relegated to second place due to the significant changes that parents face, such as lack of sleep and increased stress levels as a consequence of the high demands for their newborn baby [25,26]. For instance, Poulain et al. [22] observed that snacking was more frequent during breastfeeding than during pregnancy and after weaning. This was attributed to the extra time spent on infant care, which, in turn, led to less attention being paid to the mother’s nutritional needs.

A premature birth puts parents into a particularly worrying and stressful situation, as they must deal with the challenges of an extended hospital stay, as well as the potential death of or future consequences for their newborn child [27]. In this regard, most Level III neonatal intensive care units (NICUs) in hospitals designated as being part of the “Baby-friendly Hospital Initiative (BFHI)” follow the recommendation of the BFHI to extend neonatal wards and, therefore, greatly promote 24 h parental presence [28]. Consequently, the continuous presence of parents in the neonatal unit leads to better care for the premature infant. However, this may mean that mothers of hospitalized very premature infants (MHVPIs) do not eat properly and, consequently, that they do not achieve the specific nutritional needs of their vulnerable infants [29]. Usually, after being discharged following the birth of their child, they are excluded from the hospital meal supply. In addition, it is not uncommon for MHVPIs to develop complications after giving birth, prolonging their recovery and, therefore, their ability to feed properly.

Maternal intake has been widely investigated in healthy lactating women with healthy full-term babies [23,30,31,32]. However, MHVPIs face special circumstances, which might influence their dietary intake and, consequently, HM composition. To our knowledge, the diet and nutritional status of MHVPIs during their infant’s hospitalization, as a particular population, has rarely been investigated [23,33]. Hence, the aim of our study was to determine macronutrient, micronutrient, and FA intakes, as well as the status of nutrients in plasma, erythrocytes, and urine samples from MHVPIs. The results were compared with those of a group of HM donors (HMDs). Finally, associations were established between the MHVPIs’ nutritional intake and status and the corresponding concentration of nutrients in HM.

## 2. Materials and Methods

### 2.1. Study Population

The present study is based on data from a primarily cross-sectional designed research project carried out between May 2017 and February 2020 at the Regional Human Milk Bank “Aladina MGU” (RHMB) and the NICU of “12 de Octubre” University Hospital (Madrid, Spain). Maternal nutritional intake, plasma and erythrocyte status, and the composition of HM were investigated in the following three different populations: (1) HMDs, (2) MHVPIs, and (3) mothers following a vegetarian or vegan diet [8,10,34,35]. The hospital received the BFHI accreditation in 2014; as such, parents and authorized related persons are allowed to be with the child in the NICU 24 h a day. At the time of the study, the parents of hospitalized preterm infants were not included in the hospital food service and had to provide their own food. However, the parents’ assigned space in the NICU provided a refrigerator, a freezer, and microwaves. The available data from *n* = 15 MHVPIs and *n* = 110 HMDs were used to assess the objective of the present study. MHVPIs were recruited for this study in the hospital NICU if their child was born at less than 32 weeks of gestational age according to the definition for very preterm infants and when a sufficient supply of HM for their own child was reached, so as not to interfere in the special nutritional needs of the preterm infant. The inclusion criterion for HMDs was to be an active donor, i.e., the most recent donation was made less than two months ago. On the other hand, the exclusion criteria that were used in both groups included a breastfeeding duration of less than three weeks to ensure that lactation was well established; a drug intake that was able to modify mother’s appetite, absorption, or metabolism; malabsorptive intestinal diseases; and a lack of ability to understand the Spanish language. The Clinical Research Ethic Committee of the hospital (protocol code 15/269) approved the study, which was conducted following the Declaration of Helsinki. Written and informed consent was obtained from all participants.

### 2.2. Sample Size Calculation

The sample size was determined to meet one of the main objectives of the global study on which the present data are based: to investigate the correlations between intake, status, and the nutritional composition of HM in different groups of lactating mothers (HMDs, MHVPIs, and mothers on vegetarian or vegan diets). To ensure the generalizability of the results, at least 10 cases per each adjusted independent variable and a 5% allowance for potential dropouts were required. Therefore, a sample size of 115 lactating women was set. In this regard, we calculated the recruitment of at least 85 HMDs, 15 lactating mothers on vegetarian or vegan diets, and 15 MHVPIs.

### 2.3. Sample Collection and Context

**Figure 1:**
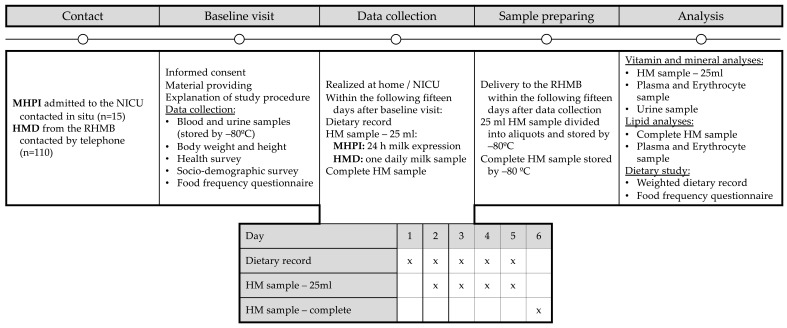
Sample collection summary. Abbreviations: HM—human milk; HMDs—human milk donors; MHVPIs—mothers of hospitalized very preterm infants; NICU—neonatal intensive care unit; RHMB—Regional Human Milk Bank “Aladina MGU”.

An extended description of the study protocol, including the study design, dietary record assessment, milk collection, and sample preparation, is provided elsewhere [35]. A further summary is also given in Figure 1. Briefly, participants were contacted, and if they agreed to participate, they were invited to a baseline visit at the RHMB to perform the following tasks: (1) to hand in a fasting urine sample of the morning, (2) to take a fasting blood sample, and (3) to provide sociodemographic, health and anthropometric data, as well as answering a short food consumption frequency questionnaire. Further information and the material used for dietary recording and milk collection were provided. The blood sample was divided into plasma and erythrocytes on the same day of collection and was frozen at −80 °C. Within the following 15 days after the baseline visit, the women filled in a weighted dietary record over 5 successive days, including a holiday, by carefully reporting all their foods, drinks, and supplements taken. The day after starting the dietary record, the women were requested to collect HM samples of 25 mL over four successive days, imitating their usual routine of daily milk expression. In this sense, as the MHVPIs carried out various pumping sessions within 24 h, we asked them to provide a small sample from each of the sessions to obtain a total volume of 25 mL daily. On the other hand, HMDs provided at least one milk expression sample per day, and, if they had more than one pumping session, they mixed the samples in the same way as the MHVPIs. The only requirement established was to carry out a full expression of one or both breasts, depending on their usual routine. Furthermore, the day after finishing the dietary record and collecting the 25 mL HM samples, one complete milk extraction was required. Within the next 15 days, the frozen HM samples stored at –20 °C in the women’s homes were transported to the RHMB to prepare them for analysis; they were subsequently frozen at −80 °C. The 25 mL HM samples were thawed and separated into aliquots of 1 mL. Hence, they underwent two freeze–thaw cycles, whereas the complete HM samples only underwent one cycle, as no further manipulation was necessary.

### 2.4. Sample Analysis

Ureta-Velasco et al., 2022, gave a detailed description of the macronutrient, micronutrient, and lipid analyses, as well as the dietary study [35].

In brief, vitamin and mineral analyses of the plasma, erythrocyte, urine, and 25 mL HM samples, were conducted by the NUTREN-Nutrigenomics Group (Department of Experimental Medicine, University of Lleida, Spain). They used the following techniques: ultra–performance liquid chromatography with tandem mass spectrometry was used to determine free thiamine, free riboflavin, nicotinamide, pantothenic acid, pyridoxal, and folic acid levels; the competitive immunoassay method was used to determine the levels of cobalamin; high-performance liquid chromatography coupled with a diode array detector was used to assess ascorbic acid levels; high-performance liquid chromatography with a florescence and ultraviolet detector was used to determine retinol, α-tocopherol, and γ-tocopherol levels; ultra-performance liquid chromatography with electrospray ionization–tandem mass spectrometry was used to evaluate vitamin D_3_ and 25(OH)D_3_ levels; and inductively coupled plasma–mass spectrometry was used to determine calcium, iodine, phosphorous and selenium levels. The NUTREN-Nutrigenomics Group also assessed hemoglobin and urine creatinine levels as well as the blood biochemistry including total cholesterol, triacylglycerol (TAG), high-density lipoprotein, and low-density lipoprotein (LDL) levels.

The Food Lipid Biomarkers and Health Group (Institute of Food Science Research (CIAL), Spanish National Research Council (CSIC) at the Autonomous University of Madrid (UAM), Spain) conducted the lipid analyses of the plasma, erythrocyte, and complete HM samples. The FA profile, determined as fatty acid methyl esters (FAMEs) for the plasma, erythrocyte, and complete HM samples, was determined in all MHVPIs and HMDs, whereas the TAG molecular species, the relative composition of phospholipids, and the lipid class profile were only determined in the HM sample of 12 MHDPs and a randomized subgroup of 20 HMDs. Gas chromatography–mass spectrometry was utilized to analyze the FAMEs, presenting them as a weight/weight percentage. The quantitation limit was 1.8 ppm, which allowed for the determination of dimethylacetal derivatives (DMAs). Lipid classes and phospholipids were quantified with high-performance liquid chromatography coupled with an evaporative light-scattering detector. On the other hand, the analysis of the TAG molecular species was conducted via gas chromatography and by using a flame ionization detector.

Finally, the HM macronutrient analysis was conducted in the complete HM sample within the RHMB using a milk analyzer with Fourier transform mid-infrared spectroscopy (MilkoScan FT2, FOSS S.A., Barcelona, Spain). For this purpose, any remaining volume of the complete HM sample used for lipid analysis was frozen and sent back to the RHMB. Hence, macronutrient analysis was carried out in HM that underwent two freeze–thaw cycles.

Diet was assessed with a short food frequency questionnaire, which was completed during the baseline visit, as well as with a 5-day weighted diet record. We used DIAL-Software^®^ (DIAL.EXE Version 3, February 2014, Alce Ingeniería, Madrid, Spain) to determine the daily energy, macronutrients, micronutrients, and lipids of the diet of the MHVPIs and HMDs, as well as their intake of eggs, meat, and fish; fruits; grains, legumes, and nuts; vegetables and greens; and dairy. Total nutrient intake included supplements and food intake. DIAL-software (DIAL.EXE Version 3, February 2014, Alce Ingeniería, Madrid, Spain) was used to calculate the Healthy Eating Index (HEI) developed by Kennedy et al. [36], which allows for an evaluation of diet quality, where “100” represents an excellent diet and “0” represents an inadequate diet. On the other hand, the food frequency questionnaire obtained information regarding the daily, weekly, or monthly consumption of the following: (1) fats and oils, (2) sweets, (3) nuts and seeds, (4) bread, pasta, rice, and others, (9) legumes, (8) cooked vegetables, (9) raw vegetables, (10) fruits, (11) eggs, (12) fish, (13) meat, and (14) milk and other dairy products. We assessed the inadequacy of mineral and vitamin intake using the harmonized average requirement (H-AR) proposed by Allen et al. [15]. Energy and protein intake was compared with the country-specific recommended intake values issued by Ortega et al. [37]. Furthermore, the IOM acceptable macronutrient distribution ranges were used to determine the inadequacy of the macronutrient percentage of total energy [38].

### 2.5. Statistical Analysis

We performed data analysis using the SPSS 26 software (IBM SPSS Statistics Inc., Chicago, IL, USA). Shapiro–Wilk tests were used to determine the continuous variables’ normality. Depending on the results, variables are presented as medians with their interquartile range (25th, 75th) or as means with their standard deviation (SD). Categorical variables are described by their absolute (*n*) and relative frequencies (%). The bivariate analyses used to assess significant differences between the MHVPI group and the HMD group in terms of baseline characteristics, dietary intake, and nutrient concentration in plasma, erythrocytes, urine, and HM were Student’s *t*-test, the Mann–Whitney U test, or Fisher’s exact test, depending on variable classification and distribution. Whenever possible, we determined the exact *p*-values, which was a computational procedure offered by the SPSS package [39] that is recommended when data are small or when the categories are unbalanced. We considered *p*-values of <0.05 to be statistically significant. Multivariate linear regression analysis was used to determine the association between the concentration of each nutrient in HM (dependent variable) and their corresponding intake and plasma, erythrocyte, and urine concentration, as well as their study group (MHVPIs vs. HMDs) (independent variables). Breastfeeding duration was included in the regression as a confounding variable. The regression was drawn in 5000 bias-corrected and accelerated bootstrap samples to overcome the problem of non-normality in most of the investigated variables. Bootstrapping, which involves the resampling and replacement of the original data, allows for the estimation of confidence intervals (CIs) without relying on assumptions about the distribution of the data. Hence, CIs were used to estimate statistical significance in the linear regression analysis, considering a 95% CI that did not include zero to be statistically significant. Initially, the nutrient concentrations in plasma and erythrocytes were introduced simultaneously into the regression model. In the case of co-correlation between the two measurements, linear regression was carried out separately for each blood component.

## 3. Results

Table 1 shows the baseline characteristics of the 15 MHVPIs. As expected, gestational weight gain was low. A high percentage had twin pregnancies and around 40% of the MHVPIs were not originally from Spain. On the other hand, most of the MHVPIs had carried out university-level studies. In Tables S1 and S2, further characteristics of the MHVPIs and HMDs can be observed. Baseline characteristics (Table S1) did not show significant differences between the two study groups except for country of origin, with MHVPIs more likely to come from a country other than Spain; the number of women currently working, which was found to be more elevated in HMDs; and a lower gestational weight gain in MHVPIs. However, because of premature birth, infant and lactation-related characteristics (Table S2) showed that the infants of MHVPIs had a lower gestational age, breastfeeding duration, birth weight, and current weight compared with the infants from HMDs, as well as significant differences in their respective weight percentiles. In the MHVPI group, higher frequencies of twin pregnancy and the use of double electric breast pumps were observed.

The pharmacological supplement consumption of MHVPIs during pregnancy and lactation are presented in Table 2. During pregnancy, the majority (93.3%) took iodine, folic acid, and vitamin B12 supplements. This consumption was maintained during lactation by 86.7% of the women at the time of the study. Vitamin A and E intake, as well as calcium supplementation intake, were low (0 to 20%) during gestation, although this increased during lactation. Additionally, Table S3 shows differences in the consumption of pharmacological supplements between the study groups. Aside from a higher daily dose of vitamin B9 during pregnancy in MHVPIs compared to HMDs, no other differences were found regarding the frequency and daily dosage of supplements during pregnancy and lactation.

Table 3 shows the daily nutrient intake, determined from the 5-day dietary record, and the prevalence of inadequate nutrient intake relative to the thresholds compiled from various sources, including the harmonized average requirement (H-AR) [15], the acceptable macronutrient distribution ranges (Institute of Medicine) [38], and Ortega et al. [37]. For comparison, the table also includes the median intake of various nutrients, based on the results of different studies from industrialized countries that examined lactating mothers of term infants, as provided by Di Maso et al. [31] in their systematic review.

Median total energy intake was 2272.6 Kcal (kilocalories), which is below the recommended dietary intake of 2430 Kcal proposed by Ortega et al. for lactating women with a sedentary lifestyle. Almost 70% of MHVPIs had an inadequate energy intake. Median daily protein intake exceeded the recommended daily protein intake of 66 g by almost 30 g. Protein intake below this value was, therefore, not observed. The median percentage of energy from carbohydrates was below its acceptable macronutrient distribution range, whereas the median percentage of energy from fat was above its acceptable macronutrient distribution ranges. Hence, we observed that more than 70% of MHVPIs showed a deficit, whereas almost 90% exceeded the ranges in relation of percentage of energy from carbohydrates and fat, respectively. The median percentages of total energy from protein and omega 3 polyunsaturated fatty acids (PUFAs) were within their respective ranges. Regardig vitamins and minerals, more than 80% of MHVPIs showed an inadequate intake for vitamin D and around 40% demonstrated an inadequate intake of iodine, as well as vitamins B8, B9, C, and E. On the other hand, except for the percentages of energy from carbohydrates, all nutrient intakes were observed to be above the medians provided by Di Maso et al. [31]. Considering study group differences, the daily nutrient intake from MHVPI’s of almost all nutrients was similar, as shown in Table S4. Disparities were only found for the percentage of caloric intake from omega 3 FAs, the intake of DHA, and, hence, the sum of eicosapentaenoic acid (EPA) and DHA intake, as well as in vitamin B12, all of which showed a higher intake in MHVPIs. Furthermore, between the two investigated groups, no differences were observed in relation to the prevalence of inadequate micronutrient intake (Table S5).

Table 4 presents the daily number of food servings from five food groups, the HEI, and the intake of dietary supplements and iodized salt, all determined from the 5-day dietary record. The table also includes the number of food servings as assessed by the food consumption frequency questionnaire. Considering the daily recommended servings for lactating mothers provided by Ortega et al. (2015) [42] and shown in Table 4, MHVPIs did not meet the recommended intake of dairy; grains, legumes, and nuts; vegetables and greens; and fruits, independent of the method used to assess food frequency (5-day dietary record or food consumption questionnaire). Furthermore, the median HEI score was 61.9 points. No differences in food servings were observed between MHVPIs and HMDs, according to the results from either the 5-day dietary record (Table S6) or the food consumption frequency questionnaire (Table S7).

Table 5 describes the FA composition (g/100 g of total fat) in the erythrocytes and plasma of 14 MHVPIs. In both samples, i.e., plasma and erythrocytes, the highest concentration of saturated fatty acids (SFAs) was observed for palmitic acid (C16:0), whereas oleic acid (C18:1 *cis*-9) was the most abundant monounsaturated fatty acid (MUFA). In erythrocytes, C20:4 presented the greatest concentration within the studied PUFAs compared with C18:2 in plasma. Several FA concentrations presented differences between HMDs and MHVPIs, as observed in Table S8. In both blood components, the concentration of DMA palmitic acid (C16:0) was higher in HMDs, whereas the *cis* vaccenic acid (C18:1 *cis*-11), dihomo-γ-linolenic acid (C20:3), DHA (C22:6), and the total omega 3 PUFAs were higher in MHVPIs. Furthermore, in erythrocytes, a higher concentration was found for DMA C18:0, arachidonic acid (20:4), and total DMAs in HMDs. On the other hand, MHVPIs presented elevated concentrations of lignoceric acid (C24:0) and linoleic acid (C18:2) in erythrocytes, as well as palmitoleic acid (C16:1 *cis*-9) and medium-chain FAs (MFCAs) in plasma.

Concentrations of nutrients and biochemical determinations in the erythrocyte, plasma, and urine samples of MHVPIs are shown in Table 6. Riboflavin deficiency in erythrocytes was 100%, when considering riboflavin concentration; however, this value was only 33.3%, when considering the erythrocyte glutathione reductase activity coefficient. In plasma, a lack of vitamin D was observed for all 15 MHVPIs and for vitamin E in 73.3%. Methylmalonic acid, a vitamin B12 deficiency marker, was used to identify that more than 70% of MHVPIs were vitamin B12 deficient. In contrast, when considering cobalamin and holotranscobalamin II concentrations, vitamin B12 deficiency was inexistent. Scurvy and a lack of vitamin A were not detected. Differences between study groups (Table S9) were observed in erythrocytes for nicotinamide, which was higher in MHVPIs, while pantothenic acid was more elevated in HMDs. On the other hand, the concentration of retinol, ascorbic acid, y-tocopherol, total cholesterol, TAG, and LDL were more elevated in MHVPIs’ plasma. However, cobalamin in plasma, as well as creatinine and phosphorus concentration in urine, were shown to be greater in HMDs.

Table 7 shows the HM lipid class profile, TAG molecular species content, and relative composition of the phospholipids from MHVPIs (*n* = 12). As expected, the most abundant lipid class was TAGs. Among these, the highest concentrations were observed in those with a molecular species of 52 carbon atoms (CNs), followed by the TAGs with 54 CNs. A comparison between MHVPIs and HMDs is shown in Table S10. The concentration of TAGs with a molecular species of 28, 30, and 32 CNs was higher in HMDs. Regarding phospholipid distribution; sphingomyelin constituted the majority in HMDs but was shown to be the lowest phospholipid in MHVPIs, where the main phospholipid groups consisted of phosphatidylcholine and phosphatidylethanolamine. All the phospholipids showed differences between the two study groups.

The FA composition (g/100 g of total fat) in HM of MHVPIs (*n* = 14) is presented in Table 8. Compared to the European references and the worldwide values [12,52], we observed that the HM concentrations in MHVPIs were lower regarding SFAs, but higher for oleic acid (C18:1 *cis*-9 (n9)), omega-6 PUFAs, and linolenic acid (C18:3). In MHVPIs, compared with HMDs (Table S11), higher concentrations were found for the PUFAs eicosadienoic acid (C20:2), dihomo-γ-linolenic acid (C20:3), and linolenic acid (C18:3), as well as for the concentrations of total PUFAs, very long-chain fatty acids (VLCFAs), and *n*-6 PUFAs, whereas lower concentrations of caproic acid (C6:0) and short-chain fatty acids (SCFAs) were found.

Table 9 shows the macronutrient, vitamin and mineral composition of the HM of MHVPIs. When compared to the references provided [14,53,54,55,56,57,58,59,60,61,62,63,64,65,66,67,68] within Table 9, MHVPIs showed higher concentrations of carbohydrates, proteins, free riboflavin, cobalamin, and iodine. Furthermore, total vitamin C, vitamer D_3_, and phosphorus levels were within their reference ranges. However, nicotinamide and calcium concentration were far below the reference values. The differences between MHVPIs and HMDs are shown in Table S12. As concerns the HM of MHVPIs, higher concentrations were determined for protein, selenium and iodine, where the difference between the two study groups was considerable.

The associations between HM nutrient concentration and nutrient intake, concentration in plasma, erythrocytes, and urine, and study group (MHVPIs–HMDs) are described in Table 10. Independent of nutrient intake, plasma, erythrocyte, and urine concentration, as well as breastfeeding duration, the MHVPI group was associated with higher HM concentration of proteins, vitamer 25(OH)D_3_, iodine, selenium, *n*-3 PUFAs, VLCFAs, and the FAs dihomo-γ-linolenic acid (C20:3) and linolenic acid (C18:3); however, it was also associated with lower concentrations of free riboflavin, dehydroascorbic acid, palmitoleic acid (C16:1 *cis*-9), and *cis* vaccenic acid (C18:1 *cis*-11). Associations were also observed between diverse HM nutrient concentrations and nutrient intake, as well as concentrations of plasma, erythrocytes, and urine, regardless of whether they were MHVPI or HMD and regardless of breastfeeding duration.

## 4. Discussion

This cross-sectional study investigated the nutrient intake and plasma, erythrocyte, and urine content of mothers whose preterm infants were admitted to the NICU of the “12 de Octubre” University Hospital in Madrid, Spain. There is a wide range of studies assessing the intake of lactating mothers [23,30,31,32] and, to a much lesser extent, their plasma, erythrocyte and urine nutritional status [2,12,13,14]. However, MHVPIs are a special population since they face circumstances related to their personal health, their preterm child’s health, and hospitalization, which goes beyond normal adaptations to the postpartum period [27].

Hence, to the best of our knowledge, this is the first study assessing not only dietary intake [33] using dietary reference intakes [15,37,38] but also by evaluating nutrient concentration in plasma, erythrocytes, and urine in MHVPIs of a large number of nutrients. Additionally, it provides a detailed analysis of their milk, including macronutrients, lipid classes, molecular species of TAGs, phospholipids, FAs, vitamins, and minerals. The results were further compared with the concentrations found in HM from donors, as they provide the second-best option to nourish a preterm infant when a mother’s own milk is not available [17,19]. Differences in HM concentration, related to prematurity rather than dietary intake, plasma and erythrocyte content, or breastfeeding duration were also assessed.

Considering the available dietary reference intakes, our results revealed that around 40% of MHVPIs showed low intakes of total energy, iodine, and vitamins B8, B9, C, D, and E, whereas an adequate percentage of energy from omega 3 PUFAs was observed. Intake of proteins was considerably higher than the recommended intake from Ortega et al. [37], although the percentage of energy from proteins was within the IOM acceptable macronutrient distribution ranges [38]. However, the percentage of energy from carbohydrates was low, while the percentage of energy from fat was high, considering the reference intake ranges.

The median energy intake of MHVPIs was 2272.6 Kcal and was, therefore, considered low in 66.7% when compared to the reference intake of 2430 Kcal from Ortega et al. [37] for Spanish lactating women with a sedentary lifestyle. Di Maso et al. [31], in their systematic review about dietary intake of lactating mothers of term infants in developed countries, summarized diverse nutrient intakes by calculating the median from the results of the included studies. The calculated median energy intake from this systematic review was 2111 kcal/day, with a range of 1411–2781 kcal, indicating that energy intake in other studies is also lower than recommended. The high percentage of energy from fat and the contrasting low Kcal percentage from carbohydrates is found to be a general occurrence in studies set in the Mediterranean area [31,69,70].

When considering the HM macronutrient content, MHVPIs showed a higher carbohydrate concentration than the reference value reported by Leghi et al. [55]; however, the lipid concentration in MHVPIs was slightly lower. The protein concentration in HM was also higher in MHVPIs compared to HMDs, with 1.36 g/dL vs. 1.19 g/dL, respectively, although both groups exceeded the reference value of 0.94 g/dL [55]. Our regression analysis showed a positive association between MHVPIs and HM protein concentration. Therefore, the higher protein concentration in HM from MHVPIs is more likely to be associated with characteristics related to prematurity, as no link with protein intake was observed.

Special attention should be paid to vitamin D intake, as in our study, over 80% of MHVPIs had an inadequate intake. Although the median intake of 6.8 µg is high compared with that of other studies [23,31], the value is below the recommended daily intake of 15 µg [37]. This finding is consistent with a review carried out by Carretero-Krug et al. [71], who determined that eight studies reported not reaching the recommended daily intake for vitamin D, while no studies reported exceeding this amount. Vitamin D deficiency was also determined in plasma for all MHVPIs and vitamer 25(OH)D_3_ showed concentrations of less than 12 ng/mL [51]. The concentration of vitamin D_3_ was within the reference range of 0.25–2.00 µg/L [64], whereas the 25(OH)D_3_ vitamer concentration (66.23 pg/mL) was substantially lower than the median value of 1100 pg/mL (44 UI/L) calculated in a meta-analysis [72], which also documented a broad range of concentrations, from 0 to 176,440 pg/mL. However, as neither the intake nor the concentration in plasma, erythrocytes, or HM were different compared with HMDs, we suppose that the vitamin D deficit is not an exclusive issue for MHVPIs. Nevertheless, the regression analysis conducted in our study showed a positive association between 25(OH)D_3_ concentrations in HM and MHVPIs as well as plasma concentration, but not with intake or erythrocyte concentration.

The increased inadequate intake of vitamin E in MHVPIs, also observed in other studies [31,73], was reflected in its plasma concentration. Furthermore, the HM concentration appeared to be lower compared to the values reported by EFSA and IOM [49,65] with 4.15 vs. 4.6 mg/L, respectively. Since no differences were found between MHVPIs and HMDs regarding vitamin E intake and α-tocopherol concentrations in plasma, erythrocytes, and HM, vitamin E deficiency should not be considered a problem exclusively for the MHVPI group but as something that also occurs in other groups of lactating mothers.

In the case of iodine, although intake was insufficient in 40% of MHVPIs, the median intake exceeded 250 µg/L and the urinary concentration was above 100 µg/L, indicating an iodine-sufficient population [74,75]. The HM concentration was above 200 µg/L, which is considered sufficient for the needs of premature infants [53,76]. In our study, the supplement intake of MHVPIs during the 5-day dietary record was over 64%, which could explain the high iodine concentrations in HM and urine. As reported in a previous study [8], iodine supplementation appears to increase HM concentration. The high percentage of inadequate intake could, therefore, be attributed to MHVPIs who did not take iodine supplements. The iodine concentration in HM was even higher in MHVPIs than in HMD. The observed positive associations between iodine HM concentration and MHPVIs as well as iodine intake confirm that prematurity but also iodine intake might affect iodine HM concentration independent from each other.

An adequate percentage of energy from omega-3 PUFAs was observed in MHVPIs, in line with the acceptable macronutrient distribution ranges established by the Institute of Medicine [38]. Both the percentage of energy derived from omega-3 PUFAs and the dietary intake of DHA (and DHA + EPA) were higher in the MHVPI group compared to the HMD group. In HM, the percentage of energy from omega-3 PUFAs and the content of α-linolenic acid were also higher in MHVPIs. In our regression analyses, both omega-3 and α-linolenic acid content in milk were positively associated with prematurity, independently of maternal intake and breastfeeding duration. Conversely, DHA content in HM was positively associated with maternal DHA intake as well as with maternal plasma and erythrocyte DHA concentrations.

Regarding the higher DHA intake in MHVPIs compared to HMDs, this was reflected in its increased concentration in plasma and erythrocytes, but not significantly in HM. Nonetheless, the median DHA concentration in HM was 0.41% for MHVPIs vs. 0.27% for HMDs. We suggest that statistical significance might have been achieved with a larger sample size of MHVPIs. These findings are surprising, as there are no differences between the study groups regarding food consumption, especially fish intake or supplementation for omega-3 FAs. However, the food consumption questionnaire and the food groups analysis provided by the 5-day dietary record did not differentiate between whitefish and oily fish; thus, a higher intake of oily fish in the MHVPI group might explain these divergences. The DHA intake found in our study was also shown to be much higher than that of the studies included in the systematic review by Di Maso et al. [31]. Regarding the plasma and erythrocyte content, Giuffrida et al. [12] reported a similar DHA concentration in erythrocytes; however, in plasma, a higher concentration was observed, at 2.36% vs. the 0.98% found in MHVPIs. Erythrocyte concentration is supposed to represent long-term intake due to its half-life of weeks or even months. Instead, plasma content might reflect short-term intake, which can be absorbed directly and transferred to HM [12,77]. Hence, a high concentration in both blood compounds might assume a regular intake and a continuous supply of DHA. It is important to mention that DHA deprivation due to preterm birth is related to a higher risk of cognitive impairment as it is an essential component for optimal brain development [78]. However, several studies suggested a daily consumption of at least 200 mg of DHA [79,80] to reach an HM concentration of 0.30% [79], which was fulfilled by the MHVPIs, who presented a median concentration of 0.41%. Regarding the special needs of low birth weight infants, the required DHA range established is 12 to 60 mg/kg/day [81]. Assuming a 1 kg preterm infant with a daily intake of 175 mL/kg/day, considering the determined lipid concentration of 3.36 g/dL found in MHVPIs in our study, we calculated a possible intake of 24.1 mg/kg/day. The calculated intake is within the recommended range but is lower than the estimated 60 mg/kg/day of the in utero requirement [78]. We showed that the HM concentration of DHA is associated with its intake and its erythrocyte status independent of prematurity and breastfeeding duration, emphasizing the influence that intake and maternal stores can have on DHA concentration in HM.

To better understand the diet of MHVPIs, we further examined their food consumption and dietary sources. MHVPIs did not achieve the recommended food servings for the five investigated food groups, according to the 5-day dietary record or food consumption questionnaire, except for the intake of eggs, meat, and fish [42]. These results are confirmed by other studies [82,83]. In this sense, Lee et al. [32] reported a transition to a less healthy maternal dietary pattern from pregnancy to postpartum, especially considering fruit and vegetable intake. On the other hand, the HEI, as a measure of dietary quality, showed a score of 61.9 points. Even though 61–70 points indicates a good diet [84], nutrition can be improved considerably. Since no differences were found in HMDs, diet quality can be considered a general issue during lactation and is not specific to MHVPIs.

As pointed out by Neville et al. [85], most studies are designed to focus principally on sampling HM, without considering the mother’s intake or plasma and erythrocyte status and, hence, fall short of investigating HM as a biological system. With the regression analysis carried out in the present study, we could determine that a diversity of macronutrients, micronutrients, and FAs in HM, independent of corresponding maternal intake, plasma and erythrocyte concentration, and breastfeeding duration, were related to prematurity. In this sense, being an MHVPI was positively associated with the HM content in terms of protein, vitamer 25(OH)D_3_, iodine, selenium, omega 3 PUFAs, VLCFAs, myristic acid, dihomo-γ-linolenic acid, and linolenic acid, and negatively associated with free riboflavin, dehydroascorbic acid, palmitoleic acid, and *cis* vaccenic acid. In most studies [86,87,88,89], differences between HM concentrations from term and preterm mothers can be observed. Preterm HM is known to have higher concentrations of certain nutrients, particularly in the first weeks postpartum. However, this composition changes rapidly and remains variable among individuals. Most studies primarily examined the concentration of macronutrients, like proteins. They found that the protein content in preterm mothers’ HM was higher, while no differences were observed for lactose and fat. There is less data available on micronutrients, but Redeuil et al. [87] reported higher levels of vitamins B1, B2, B3, B6, and B9, along with lower levels of vitamins A and E in the HM of mothers who delivered prematurely. Nevertheless, the possibility that some of these associations might be based on maternal intake rather than preterm and term delivery cannot be ruled out. Many factors related to preterm delivery can influence HM composition, such as shortening the prenatal preparation of the mammary gland, immature oral feeding skills of the infant, and the use of alternative milk removal methods [18]. The shortened gestation period might be a further reason [18]. Maternal body fat is accumulated during the first two trimesters of gestation, whereas in the last trimester, a transfer of fat occurs to fill the stores of the infant’s adipose tissue [90]. Preterm delivery interrupts this process; therefore, preterm infants are deprived of maternal supply, and the amount of maternal fat that is not used can be used as a reserve of FAs during breastfeeding. To the best of our knowledge, this is the first study that attributes changes in HM concentration to MHVPIs independent of maternal diet. However, the small number of MHVPIs and the great heterogeneity in the characteristics of both study groups should be taken into account when interpreting these results. Additionally, associations were also found in the regression analysis for nutrient intake, as well as nutrient plasma and erythrocyte status with HM concentration for several vitamers, minerals, and FAs independent of the study group and breastfeeding duration.

Adequate nutritional intake is important for lactating women, particularly for MHVPIs and HMDs, as they provide their milk to infants who have nutritional deficiencies and an immature organ system, which makes adequate absorption, utilization, and retention of nutrients difficult [18]. Very preterm infants have markedly higher nutritional requirements, especially for protein, calcium, phosphorus, DHA, and several micronutrients, due to their limited body stores and the need to support rapid growth and organ development [91,92]. Even though HM provides immunological and gastrointestinal benefits, its native nutrient content is often insufficient to meet these elevated demands, especially when donor milk is used. Variability in the composition of maternal or donor milk can lead to suboptimal nutrient intake if not properly addressed through individualized fortification strategies. Inadequate intake during this critical window may negatively impact neurodevelopment, bone mineralization, and growth outcomes, as shown in previous studies [93,94,95,96,97]. Furthermore, some nutrients naturally present in milk (e.g., DHA, iron, and vitamin B12) may have better bioavailability than those added through fortification or supplementation. Despite the benefits of HM, its nutrient content alone is often insufficient to meet the elevated needs of preterm neonates, particularly for protein, calcium, phosphorus, and other micronutrients. Recent ESPGHAN guidelines support the routine use of multicomponent fortifiers rather than isolated nutrient supplements [98]. However, the biodisponibility of nutrients may differ depending on whether they are naturally present in milk or added through fortifiers. This is partly because HM contains bioactive components that protect nutrients during digestion and enhance their absorption and utilization, either through natural binding to specific transport proteins or through their incorporation into milk fat globules, both of which support efficient nutrient uptake and of tissue distribution [99]. For example, vitamin B12 in HM is bound to haptocorrin, which protects it during digestion and enhances absorption [100]. In the case of DHA, it has been shown that its incorporation into infant tissues is more efficient when derived from HM compared to direct supplementation with isolated fish oil capsules [101,102,103]. These findings reinforce the importance of preserving and optimizing the nutritional quality of HM, particularly in vulnerable populations, such as preterm infants. For this reason, it becomes especially important to study the impact of maternal diet on milk composition and to implement strategies aimed at improving the nutritional status of lactating women, with the goal of optimizing the quality of milk available to preterm infants.

Our study showed that the nutritional intake of MHVPIs did not differ greatly from the intake of HMDs or from the intake of breastfeeding mothers of term infants, considering the results of Di Maso et al. [31]. Hence, the nutritional deficiencies found in MHVPIs are also present in other lactating populations. There are ways to improve the diets of MHVPIs. For example, the total energy requirements of MHVPIs were not met, and this gap could be closed by a higher intake of fruits, vegetables, whole grains, legumes, and nuts, which would simultaneously add important micronutrients to their diet and, thus, reduce vitamin and mineral deficiencies. In addition, the percentage of energy from carbohydrates was below the acceptable macronutrient distribution ranges, while the percentage of energy from fat was above the corresponding range. Including the above-mentioned food groups in the diet of MHVPIs could compensate for this imbalance. On the other hand, the realized regression analysis confirmed associations with HM concentration not only in terms of study groups but also with intake and nutritional status, which further emphasizes the importance of both aspects in lactating mothers.

Hence, dietary education should be offered to MHVPIs. Professionals, such as dietitians, should provide supportive educational and intervention programs that explain the importance of nutrition for MHVPIs themselves and their premature infants. Just as NICUs offer workshops and training courses to promote successful breastfeeding [104], similar efforts could be made to successfully adapt the diet of MHVPIs to meet their nutritional and dietary needs, as well as those of their premature infants [105]. On the other hand, MHVPIs should be included in hospital meal services to facilitate their access to healthy food. Although they are not hospitalized patients, they are still the nutritional care keepers of their hospitalized preterm children [18,85]. Furthermore, nutrient supplementation for MHVPIs should also be considered, as several studies have observed that this could increase the concentration of several nutrients in HM [3,8,10]. Currently, in the region of Madrid, free supplementation is only offered for the nutrients iodine, vitamin B9, and vitamin B12, which should be extended.

This study has several limitations. First of all, the sample size is small in the MHVPI group. Still, the study provides a very comprehensive assessment of their dietary conditions, evaluating nutrient intake, including food consumption and dietary sources but also nutrient concentrations in plasma, erythrocytes, and urine, along with information about HM composition. As concerns the statistical bivariate and multivariate analysis, the small sample size and unbalanced categories were overcome to some extent with such tools as bootstrapping and the determination of the exact *p*-values rather than the use of approximations. Nevertheless, the results should be confirmed by further studies with a more representative sample size.

Secondly, the sample was not randomly selected. Participants were enrolled through opportunity sampling, and their inclusion was based on voluntary consent. For example, it is plausible that mothers with higher educational levels were more likely to understand the study objectives, felt confident in engaging with the research process, and, therefore, were more inclined to participate [106]. On the other hand, the inclusion of MHVPIs in the study depended on the milk supply, as it was essential for us to assure that the MHVPIs who participated in our study were able to express enough milk to fulfill the needs of their preterm infants before delivering HM for study purposes, especially as a full expression was required. Mothers who could not express enough milk did not participate in the study. Those who participated joined the study at different postpartum time points. Both the amount of HM and the duration of breastfeeding affect HM composition. In addition, we would like to emphasize that the studied HM in both groups was mature milk, as one of the inclusion criteria was that mothers had been lactating for at least three weeks postpartum.

Regarding reporting bias, the 5-day dietary record, when administered with appropriate instructions and a sufficient number of observation days, has been shown to have high validity and precision, and is often considered a reference method in validation studies. One of the main strengths of the 5-day dietary record is that foods and beverages are recorded at the time of consumption, which minimizes memory-related omissions. Additionally, it allows for the registration of dietary intake as actually consumed, including preparation details and portion sizes, especially when weighed records are used. Nonetheless, we acknowledge the limitations of this method. These include potential social desirability bias, where participants may underreport foods perceived as less healthy; respondent burden, which may affect compliance and accuracy; and altered eating behaviors, where participants modify their intake to simplify the recording process. Some individuals may also face challenges in accurately describing portion sizes or recording all items consumed. Furthermore, increasing the number of recording days may reduce data quality, and the method entails a high cost of data processing and coding.

Untreated donor HM was examined, and it is uncertain whether the observed results—particularly the associations from the regression analysis—would persist if pasteurized donor HM were studied. However, preservation through Holder pasteurization appears to primarily affect bioactive and immunomodulatory components rather than nutrients [107]. A significant decrease was observed in vitamin B6, C, and folic acid due to pasteurization, while other vitamins and fatty acids remained unaffected [108].

Finally, while a large number of nutrients were considered in this study, gaps remain for many nutrients that still need to be addressed. This applies, for example, for choline intake and the three water-soluble forms of choline (free choline, phosphocholine, and glycerophosphocholine) in HM, which are essential for lipid synthesis, neurotransmission, and methylation, and are, therefore, crucial for normal growth in infants.

Nevertheless, the wide range of nutrients examined, the detailed dietary assessment, the biochemical analysis of plasma, erythrocytes, and urine, and the multiple milk samples collected from a rarely studied group of breastfeeding women are strengths that set our study apart from others.

## 5. Conclusions

In conclusion, we presented the first study to comprehensively assess the nutrient intake, nutritional status, and HM composition in MHVPIs. This population deals with a particularly worrying and stressful situation involving their personal health, their vulnerable newborn child’s health, and the challenges of hospitalization, and all these factors can lead to an inadequate diet. In addition, we provided further insights into the study of HM by relating its composition with prematurity independent of maternal nutrient intake and plasma, erythrocyte, and urine status. Regarding the results of the present study, the diet evaluation of MHVPIs, considering dietary reference intakes, showed that a high proportion had inadequate nutrient intakes for total energy, as well as iodine and vitamins B8, B9, C, D, and E. Moreover, a high intake of protein was observed. The percentage of energy from carbohydrates was low, whereas the percentage of energy from fat was high, considering the IOM acceptable macronutrient distribution ranges. However, we found that the diet of MHVPIs did not differ substantially from that of HMDs, which was confirmed by several studies considering the mothers of term infants. Hence, deficient food consumption of several nutrients did not seem to be related exclusively to MHVPIs but appeared to be population based (lactating women). However, the consequences of an insufficient diet on HM composition are more serious for preterm infants and, therefore, should be addressed to improve preterm infant outcomes. On the other hand, associations were found between HM concentrations of protein, several micronutrients, and FAs and the study group (MHVPIs vs. HMDs), independent of maternal diet. Furthermore, maternal characteristics revealed that premature birth could modify HM composition.

## Figures and Tables

**Table 1:** Baseline characteristics of mothers with hospitalized very preterm infants (*n* = 15) and their breastfed infants.

Characteristics	
Age (years)	35.7 (6.6); 26.1–51.5
Pre-pregnancy BMI (kg/m^2^)	24.5 (20.1, 29.8); 17.6–36.2
Current BMI (kg/m^2^)	24.7 (22.5, 29.1); 18.1–35.7
Gestational weight gain (kg)	7.0 (3.5, 10.0); 0.0–12.0
Number of children	
1	7 (46.7)
2	6 (40.0)
≥3	2 (13.3)
Country of origin: Spain	9 (60.2)
Education level	
Secondary studies	1 (6.7)
Technical studies	3 (20.0)
University studies	11 (73.3)
Smoking before pregnancy (yes)	3 (20.0)
Passive smoking (yes)	3 (20.0)
Alcohol consumption	
Prior to pregnancy (yes)	7 (46.7)
During pregnancy (yes)	0 (0.0)
Currently (yes)	0 (0.0)
Sex (girl) ^1^	13 (65.0)
Twin pregnancy ^2^	6 (40.0)
Gestational age (weeks)	26^+0^ (25^+0^, 27^+2^); 23^+5^–30^+3^
Post-menstrual age (weeks)	34^+4^ (31^+6^, 35^+3^); 27^+4^–41^+4^
Birth weight (g) ^1^	735.0 (642.5, 990.0); 535–1750
Breastfeeding duration (months)	1.7 (1.1, 2.5); 0.7–3.2
Sum of child breastfeeding times and pump sessions per day	
5–10	12 (80.0)
>10	3 (20.0)
Twin breastfeeding (yes)	4 (26.7)
Milk expression type ^3^	
Manual	0 (0.0)
Mechanical breast pump	1 (6.7)
Simple electric breast pump	8 (53.3)
Double electric breast pump	9 (60.0)
Season during the study	
Spring	2 (13.3)
Summer	2 (13.3)
Autumn	3 (20.0)
Winter	8 (53.3)

Normally distributed continuous variables: mean (standard deviation). Non-normally distributed continuous variables: median (25th, 75th percentile). Qualitative variables: absolute (*n*) and relative frequencies (%). Ranges for quantitative variables are shown after the semicolons. Abbreviations: BMI—body mass index. ^1^ *n* = 20, because of twin birth. ^2^ One of the mothers with hospitalized preterm infants from a twin pregnancy lost one of the children before giving birth. ^3^ Categories do not exclude each other.

**Table 2:** Frequency of intake and daily doses of pharmacological dietary supplements during pregnancy and lactation in mothers of hospitalized very preterm infants (*n* = 15).

		Frequency	Daily Dose
		*n* (%)	Median (25th, 75th); Range
Vitamin A, µg	Pregnancy	2 (13.3)	700.0 (-); 700.0–700.0
	Lactation	8 (53.3)	
	Previously ^1,2^	1 (12.5)	800.0 (-); 800.0–800.0
	Currently ^1,3^	7 (87.5)	800.0 (400.0, 800.0); 400.0–1000.0
Vitamin D, µg	Pregnancy	7 (46.7)	10.0 (5.0, 15.0); 5.0–15.0
	Lactation	8 (53.3)	
	Previously	1 (12.5)	5 (-); 5.0–5.0
	Currently	7 (87.5)	5.0 (2.5, 5.0); 2.5–5.0
Vitamin E, mg	Pregnancy	3 (20.0)	12.0 (5.0, -); 5.0–12.0
	Lactation	8 (53.3)	
	Previously	1 (12.5)	12.0 (-); 12.0–12.0
	Currently	7 (87.5)	12.0 (6.0, 12.0); 6.0–15.0
Vitamin C, mg	Pregnancy	7 (46.7)	40.0 (40.0, 80.0); 40.0–80.0
	Lactation	8 (53.3)	
	Previously	1 (12.5)	80.0 (-); 80.0–80.0
	Currently	7 (87.5)	80.0 (40.0, 80.0); 40.0–100.0
Vitamin B1, mg	Pregnancy	7 (46.7)	1.1 (-); 1.1–1.1
	Lactation	8 (53.3)	
	Previously	1 (12.5)	1.1 (-); 1.1–1.1
	Currently	7 (87.5	1.1 (0.8, 1.1); 0.6–1.1
Vitamin B2, mg	Pregnancy	7 (46.7)	1.4 (-); 1.4–1.4
	Lactation	8 (53.3)	
	Previously	1 (12.5)	1.4 (-); 1.4–1.4
	Currently	7 (87.5	1.4 (1.1, 1.4); 0.7–1.6
Vitamin B3, mg	Pregnancy	7 (46.7)	16.0 (-); 16.0–16.0
	Lactation	8 (53.3)	
	Previously	1 (12.5)	16.0 (-); 16.0–16.0
	Currently	7 (87.5	16.0 (8.0, 16.0); 8.0–16.0
Vitamin B5, mg	Pregnancy	7 (46.7)	6.0 (-); 6.0–6.0
	Lactation	8 (53.3)	
	Previously	1 (12.5)	6.0 (-); 6.0–6.0
	Currently	7 (87.5	6.0 (3.0, 6.0); 3.0–6.0
Vitamin B6, mg	Pregnancy	8 (53.3)	1.4 (1.4, 1.4); 1.4–5.0
	Lactation	8 (53.3)	
	Previously	1 (12.5)	1.4 (-); 1.4–1.4
	Currently	7 (87.5	1.4 (0.7, 1.4); 0.7–2.0
Vitamin B8, µg	Pregnancy	7 (46.7)	50.0 (-); 50.0–50.0
	Lactation	8 (53.3)	
	Previously	1 (12.5)	50.0 (-); 50.0–50.0
	Currently	7 (87.5	50.0 (25.0, 50.0); 25–50
Vitamin B9, µg	Pregnancy	14 (93.3)	400.0 (400.0, 1305.3); 400.0–4233.0
	Lactation	13 (86.7)	
	Previously ^1^	0.(0.0)	-
	Currently ^2^	13 (100.0)	300.0 (200.0, 400.0); 100.0–400.0
Vitamin B12, µg	Pregnancy	14 (93.3)	2.3 (2.0, 2.5); 2.0–2.5
	Lactation	13 (86.7)	
	Previously	0 (0.0)	-
	Currently	13 (100.0)	2.0 (2.0, 2.5); 1.3–2.5
Calcium, mg	Pregnancy	0 (0.0)	-
	Lactation	7 (46.7)	
	Previously	1 (14.3)	200 (-); 200–200
	Currently	6 (85.7)	200.0 (100.0, 200.0); 100.0–200.0
Iodine, µg	Pregnancy	14 (93.3)	200.0 (200.0, 200.0); 150.0–200.0
	Lactation	13 (86.7)	
	Previously	0 (0.0)	-
	Currently	13 (100.0)	200.0 (200.0, 200.0); 100.0–200.0
Iron, mg	Pregnancy	8 (53.3)	30.0 (17.5, 41.3); 14.0–80.0
	Lactation	10 (66.7)	
	Previously	1 (10.0)	80 (-); 80.0–80.0
	Currently	9 (90.0)	14.0 (14.0, 30.5); 7.0–114.0
Selenium, µg	Pregnancy	7 (46.7)	55.0 (-); 55.0–55.0
	Lactation	8 (53.3)	
	Previously	1 (12.5)	20 (-); 20.0–20.0
	Currently	7 (87.5)	20.0 (15.0, 55.0); 10.0–55.0
Zinc, mg	Pregnancy	7 (46.7)	10.0 (-); 10.0–10.0
	Lactation	8 (53.3)	
	Previously	1 (12.5)	10.0 (-); 10.0–10.0
	Currently	7 (87.5)	10.50 (5.0, 10.0); 5.0–10.0
Omega 3, g	Pregnancy	7 (46.7)	0.20 (0.16, 0.20); 0.16–0.22
	Lactation	8 (53.3)	
	Previously	1 (12.5)	0.24 8–9; 0.24–0.24
	Currently	7 (87.5)	0.24 (0.12, 0.24); 0.12–0.24

^1^ % depends on the frequency of the intake of pharmacological dietary supplements during lactation. ^2^ Previously: at the time of the study, women had stopped taking supplements during lactation. ^3^ Currently: at the time of the study, women still took supplements.

**Table 3:** Daily nutrient intake determined according to the 5-day dietary record of mothers of hospitalized very preterm infants (*n* = 15) and the prevalence of inadequate nutrient intake, regarding the harmonized average requirement (H-AR) proposed by Allen et al. [15], recommended daily intakes from Ortega et al. [37], and the Acceptable Macronutrient Distribution Ranges (AMDRs) from the Institute of Medicine [38].

	Daily Nutrient Intake	^1^	Cut-Off Points ^2^	Di Maso et al. [31] ^3^ Median (25th, 75th)
Energy (Kcal)	2272.6 (1893.8, 2734.6)	10 (66.7)	2430	2111 (1949, 2325)
Protein (g)	93.7 (72.4, 105.6)	0.0 (0.0)	66	85.4 (78.9, 91.5)
Total fat (g)	104.0 (79.1, 113.5)			82.4 (62.7, 97.0)
Saturated fat (g)	31.1 (26.9, 39.7)			33.7 (-)
Monounsaturated fat (g)	40.4 (30.4, 48.0)			32.8 (-)
Polyunsaturated fat (g)	18.8 (12.4, 20.3)			12.7 (-)
PUFAs/SFAs	0.59 (0.50, 0.72)			
(PUFAs + MUFAs)/SFAs	2.0 (1.7, 2.2)			
Kcal from carbohydrate (%)	42.8 (4.2)	11 (73.3) ^4^	45–65	49.5 (41.1–65.5)
Kcal from protein (%)	17.6 (15.7, 18.5)	0.0 (0.0)	10–35	
Kcal from fat (%)	39.1 (6.0)	13 (86.7) ^5^	20–35	
Kcal from saturated fat (%)	12.6 (2.5)			
Kcal from polyunsaturated fat (%)	6.7 (5.9, 8.3)			
Kcal from monounsaturated fat (%)	15.8 (14.2, 18.9)			
Kcal from *n*-3 fatty acids (%)	0.95 (0.82, 1.22)	4 (26.7) ^5^	0.6–1.2	
*n*-6 fatty acids (g)	15.6 (9.8, 18.3)			12.1 (-)
*n*-3 fatty acids (g)	2.2 (1.7, 3.1)			1.8 (-)
*n*-6/*n*-3 fatty acids	7.3 (6.2, 8.9)			
Myristic acid (C14:0) (g)	2.9 (1.9, 3.6)			
Palmitic acid (C16:0) (g)	15.4 (13.6, 20.0)			
Palmitoleic acid (C16:1 n7) (g)	1.5 (0.5)			
Stearic acid (C18:0) (g)	6.7 (5.1, 8.9)			
Oleic acid (C18:1n9c) (g)	38.3 (27.5, 44.5)			
Linoleic acid (C18:2n6c) (g)	15.5 (9.7, 18.2)			9.9 (-)
Linolenic acid (C18:3n3) (g)	1.7 (1.2, 2.3)			1.2 (-)
EPA (C20:5n3) (g)	0.13 (0.09, 0.23)			0.04 (-)
Docosapentaenoic acid (C22:5n3) (g)	0.03 (0.02, 0.08)			
DHA (C22:6n3) (g)	0.45 (0.33, 0.56)			0.09 (-)
EPA + DHA (g)	0.58 (0.47, 0.82			
*Trans* fatty acids (g)	0.46 (0.40, 0.61)			
Cholesterol (mg)	343.6 (294.2, 438.2)			276 (-)
Cholesterol (mg/1000 Kcal)	147.2 (135.3, 189.1)			
Thiamine (B1) (mg)	1.9 (1.3, 2.8)	3 (20.0)	1.2	1.6 (-)
Riboflavin (B2) (mg)	2.6 (2.3, 3.6)	2 (13.3)	1.7	2.0(-)
Niacin (B3) (mg)	47.3 (30.9, 60.9)	0 (0.0)	13	20.6 (-)
Pantothenic acid (B5) (mg)	8.6 (5.4, 11.4)	5 (33.3)	5.6	
Pyridoxine (B6) (mg)	2.7 (2.2, 3.6)	1 (6.7)	1.4	2.5 (-)
Biotin (B8) (µg)	43.7 (31.5, 77.7)	6 (40.0)	36	
Folate food + folic acid (B9) (µg)	478.4 (278.6, 678.2)	6 (40.0)	380	
Cobalamin (B12) (µg)	7.5 (6.8, 8.5)	0 (0.0)	2.4	
Vitamin A (µg)	1441.2 (1107.4, 1981.2)	2 (13.3)	1020	1132.0 (-)
Vitamin C (mg)	141.2 (89.2, 252.2)	8 (53.3)	145	122.0(-)
Vitamin D (µg)	6.8 (3.5, 7.8)	13 (86.7)	10	3.1 (-)
Vitamin E (µg)	17.5 (11.8, 24.4)	7 (46.7)	16	10.0 (-)
Calcium (mg)	1152.4 (918.0, 1429.2)	3 (20.0)	750/860 ^6^	1001.0 (-)
Iodine (µg)	259.4 (141.6, 334.6)	6 (40.0)	209	
Iron (mg)	22.3 (12.5, 32.7)	1 (6.7)	11.2 ^7^	14.8 (-)
Phosphorus (mg)	1675.8 (1388.0, 1947.4)	0 (0.0)	580	1465.0 (-)
Selenium (µg)	128.8 (98.9, 139.4)	1 (6.7)	59	
Zinc (mg)	13.4 (9.5, 20.7)	5 (33.3)	10 ^8^	10.0 (-)

Daily nutrient intake: Normally distributed continuous variable: mean (standard deviation). Non-normally distributed continuous variable: median (median (25th, 75th percentile)). ^1^: Qualitative variable: absolute (*n*) and relative frequencies (%). ^1^ The prevalence of MHVPIs whose intake was found to be outside the cut-off points. ^2^ Cut-off points compiled by different sources, including harmonized average requirement (H-AR), AMDRs (Institute of Medicine), and Ortega et al. ^3^ Medians determined according to studies from developed countries and provided by Di Maso et al. [31] in his systematic review. ^4^ Below the inferior limit of the reference range. ^5^ Above the superior limit of the reference range. ^6^ In the case of calcium, the H-AR proposed for lactating mothers is 860 mg if they are ≤18–30 years and 750 mg if 31–50 years. We calculated inadequate nutrient intake corresponding to the age of each woman. ^7^ In the case of iron, the H-AR values proposed depend on absorption (high, moderate, and low). Low absorption is characteristic of a plant-based diet. None of the investigated mothers of hospitalized very preterm infants followed a plant-based diet; therefore, we assumed a moderate absorption. ^8^ In the case of zinc, the H-AR values proposed depend on phytate intake (refined, semi-refined, semi-unrefined, and unrefined). Due to the abandonment of the traditional Mediterranean dietary patterns in the Spanish population [40,41], we assumed a semi-refined diet. Abbreviations: DHA—docosahexaenoic acid, EPA—eicosapentaenoic acid, Kcal—kilocalories, MUFAs—monounsaturated fatty acids, PUFAs—polyunsaturated fatty acids, SFAs—saturated fatty acids.

**Table 4:** Daily number of food servings, HEI, and intake of supplements and iodized salt determined using the 5-day dietary record. Number of food portions determined using the food consumption frequency questionnaire in 15 mothers of hospitalized very preterm infants.

5-Day Dietary Record		Recom. ^1^	Food Consumption Frequency Questionnaire	
Dairy (servings/day)	2.6 (1.8, 3.1)	≥4	Milk (servings/day)	1.5 (0.6, 2.2)
			Other dairy products (servings/day)	2.3 (0.8, 3.9)
Eggs, meat, and fish (servings/day)	3.1 (1.0)	2–3	Meats and derivatives (servings/day)	0.9 (0.4, 1.3)
			Fish (servings/week)	2.2 (1.1, 2.9)
			Eggs (servings/week)	2.8 (2.0, 3.5)
Fruits (servings/day)	1.9 (0.8, 2.7)	≥3	Fruits (servings/day)	1.3 (0.6, 3.1)
Vegetables and greens (servings/day)	3.2 (2.1, 3.8)	≥4	Raw vegetables (servings/day)	0.5 (0.2, 0.9)
			Cooked vegetables (servings/day)	0.9 (0.6, 0.9)
Grains, legumes, and nuts (servings/day)	5.1 (1.8)	≥7	Legumes (servings/week)	2.9 (1.4, 4.3)
			Bread (servings/day)	1.0 (0.4, 1.6)
			Pasta, rice, other grains (servings/week)	5.4 (3.6, 11.3)
			Nuts (servings/week)	7.0 (0.4, 14.0)
			Oils and fats (servings/day) ^2^	2.0 (0.9, 2.6)
			Sweets (grams/week) ^3^	3.9 (0.8, 5.6)
HEI	61.9 (8.3)			
Supplement intake (yes)	9 (64.3)			
Iodized salt intake (yes)	10 (66.7)			
Salt (g/day)	1.0 (0.6)			

Normally distributed continuous variable: mean (standard deviation). Non-normally distributed continuous variable: median (25th, 75th percentile). Qualitative variable: absolute (*n*) and relative frequencies (%). ^1^ Number of recommended daily servings of food for lactating women [42]. ^2^ *n* = 14. ^3^ *n* = 7. Abbreviations: HEI—Healthy Eating Index, Recom.—recommendations.

**Table 5:** Fatty acid composition (g/100 g of total fat) in the erythrocyte and plasma of mothers of hospitalized very preterm infants (*n* = 14).

Fatty Acid (%)	Common Name	Erythrocytes	Plasma
**SFAs**			
C14:0	Myristic	0.12 (0.04)	0.41 (0.19, 0.49)
C15:0	Pentadeccanoic	-	0.05 (0.04, 0.06)
DMA C16:0	Dimethylacetal C16:0	1.96 (0.20)	0.16 (0.12, 0.19)
C16:0	Palmitic	21.77 (1.44)	20.41 (19.26, 21.28)
DMA C18:0	Dimethylacetal C18:0	3.01 (0.25)	0.07 (0.05, 0.09)
C18:0	Stearic	19.57 (18.01, 21.08)	6.21 (5.76, 6.61)
C24:0	Lignoceric	3.14 (2.19, 3.95)	-
**MUFAs**			
C16:1 *cis*-9 (n7)	Palmitoleic	-	0.55 (0.43, 0.77)
C17:1	Margaroleic	0.34 (0.26, 0.38)	-
C18:1 *cis*-11 (n7)	*Cis* vaccenic	0.27 (0.24, 0.32)	0.52 (0.44, 0.66)
C18:1 *cis*-9 (n9)	Oleic	11.86 (1.80)	18.13 (16.21, 21.42)
***n*** **-6 PUFAs**			
C18:2 (n6)	Linoleic	9.31 (1.74)	40.56 (35.27, 44.46)
C20:3 (n6)	Dihomo-γ-linolenic	1.51 (1.24, 2.29)	1.39 (1.19, 1.82)
C20:4 (n6)	Arachidonic	22.14 (4.25)	10.42 (2.96)
***n*** **-3 PUFAs**			
C20:5 (n3)	Eicosapentaenoic	0.00 (0.00, 0.31)	0.17 (0.14, 0.36)
C22:5 (n3)	Docosapentaenoic	0.83 (0.25)	-
C22:6 (n3)	Docosahexaenoic	3.97 (1.23)	0.98 (0.87, 1.52)
**Fatty Acid Families**			
DMAs		4.97 (0.36)	0.22 (0.17, 0.29)
SFAs		44.43 (2.52)	26.91 (25.76, 28.34)
MUFAs		12.49 (1.90)	19.07 (17.11, 22.74
PUFAs		36.86 (35.62, 42.09)	54.77 (48.89, 56.92)
MCFAs		0.12 (0.04)	0.46 (0.26, 0.55)
LCFAs		62.89 (4.74)	86.10 (3.09)
VLCFAs		28.81 (4.04)	12.90 (10.10, 15.77)
*n*-6 PUFAs		33.20 (3.36)	53.11 (48.01, 55.47)
*n*-3 PUFAs		4.92 (1.47)	1.26 (1.03, 1.70)
*n*-6 PUFAs/*n*-3 PUFAs		5.70 (5.37, 9.43)	40.16 (32.92, 51.41)

Normally distributed continuous variable: mean (standard deviation). Non-normally distributed continuous variable: median (25th, 75th percentile). Abbreviations: DMA—dimethylacetal, LCFAs—long-chain fatty acids, MCFAs—medium-chain fatty acids, MUFAs—monounsaturated fatty acids, PUFAs—polyunsaturated fatty acids, SCFAs—short-chain fatty acids, SFAs—saturated fatty acids, VLCFAs—very long-chain fatty acids.

**Table 6:** Concentrations of nutrients and biochemical determinations in the erythrocyte, plasma, and urine of mothers of hospitalized very preterm infants (*n* = 15).

	Unit ^1^		Cut-Offs	*n* (%)
		**Erythrocytes**		
Hemoglobin	g/dL	25.13 (21.60, 26.23)		
EGRAC ^2^		1.09 (0.17)	Normal EGRAC < 1.2 [43]	6 (66.7)
Riboflavin, B2	ng/L	948.00 (499.30, 1195.80)		
	nM	2.52 (1.33, 3.18)	Riboflavin deficiency < 170 [44]	15 (100.0)
	ng/hHb	3.51 (1.99, 5.00)		
Nicotinamide, B3	µg/L	7572.00 (6411.10, 8684.30)		
	µM	62.00 (52.50, 71.11)		
	µg/g Hb	32.27 (28.12, 37.24)		
Pantothenic acid, B5	µg/L	12.20 (6.10, 16.20)		
	nM	55.6 (27.8, 73.9)		
	mg/g Hb	48.22 (26.87, 73.60)		
Pyridoxamine, B6	µg/L	497.00 (454.70, 606.70)		
	µM	2.94 (2.69, 3.59)		
	µg/g Hb	2.14 (1.83, 2.87)		
		**Plasma**		
Thiamin, B1	µg/L	0.32 (0.14, 1.08)		
	nM	0.95 (0.42, 3.20)		
Riboflavin, B2	µg/L	19.35 (15.46, 34.70)		
	nM	51.41 (41.08, 82.20)	Riboflavin deficiency < 6.7 [45]	0 (0.0)
Nicotinamide, B3	µg/L	4.82 (2.87, 5.80)		
	nM	39.46 (23.50, 47.49)		
Pantothenic acid, B5	µg/L	132.98 (88.60, 159.31)		
	nM	606.50 (404.10, 726.60)		
Pyridoxine, B6	µg/L	151.49 (120.78, 162.17)		
	nM	895.31 (713.81, 958.42)		
Pyridoxamine, B6	µg/L	258.16 (234.05, 280.06)		
	nM	1525.73 (1383.24, 1655.15)		
Folic acid, B9	µg/L	2.36 (1.77, 3.27)		
	nM	5.36 (4.02, 7.43)		10 (66.7)
Cobalamin, B12	pM	409.00 (342.00, 524.00)	Normal B12 > 221 [46]	15 (100.0)
Holotranscobalamin II	pM	176.50 (131.00, 214.00)	B12 depletion < 35 [47]	0 (0.0)
Homocysteine	µg/M	11.41 (3.73)	Elevated Homocysteine > 13 [48]	6 (40.0)
Ascorbic acid, C	µM	65.90 (44.50, 92.90)	Scurvy < 11 [49]	0 (0.0)
Retinol, A	µg/dL	60.76 (11.88)	Vitamin A deficiency < 20 [50]	0 (0.0)
	µM	2.12 (0.41)		
25(OH)D_3_, D	ng/mL	7.06 (4.78, 8.87)	Vitamin D deficiency < 12 [51]	15 (100.0)
	nM	17.62 (11.93, 22.15)		
1,25(OH)D_3_, D	pg/mL	94.40 (71.60, 318.41)		
	pM	226.60 (171.87, 764.31)		
α-tocopherol	µg/dL	248.90 (187.70, 914.10)	Vitamin E deficiency < 500 [49]	11 (73.3)
	µM	5.78 (4.36, 21.22)		
γ-tocopherol	µg/dL	50.50 (42.50, 68.20)		
Total cholesterol	mg/dL	219.50 (183.20, 257.80)	Hypercholesterolemia > 240 [51]	6 (40.0)
	mM	5.69 (4.74, 6.68)		
Triacylglycerols	mg/dL	57.40 (46.70, 92.30)	Hypertriglyceridemia > 200 [51]	1 (6.7)
HDL	mg/dL	63.20 (59.10, 67.50)	Low HDL levels < 40 [51]	0 (0.0)
	mM	1.64 (1.53, 1.75)		
LDL	mg/dL	118.30 (109.40, 154.30)	High LDL levels > 160 [51]	1 (6.7)
	mM	3.06 (2.83, 3.99)		
		**Urine**		
Cr	mg/dL	90.78 (44.64)		
Methylmalonic acid	mg/L	4.59 (2.77, 7.52)	B12 deficiency marker > 4 [47]	11 (73.3)
	µg/mg Cr	4.88 (3.76, 8.61)		
Iodine	µg/L	106.04 (67.93, 141.60)		
	µg/mg Cr	0.14 (0.10, 0.17)		
Sodium	mg/L	2898.89 (1450.41)		
	mg/mg Cr	3.51 (2.07,5.18)		
Calcium	mg/L	91.90 (37.00, 151.00)		
	mg/mg Cr	0076 (0.59, 0.84)		
Phosphorus	mg/L	717.70 (322.40, 875.44)		
	mg/mg Cr	0.11 (0.07, 0.18)		

Normally distributed continuous variable: mean (standard deviation). Non-normally distributed continuous variable: median (25th, 75th percentile). ^1^ The units of our results have been converted to the international system. ^2^ *n* = 9. Abbreviations: Cr—creatinine, EGRAC—erythrocyte glutathione reductase activity coefficient, HDL—high-density lipoprotein, LDL—low-density lipoprotein, M—molar, *n*—number of samples, SE—standard error.

**Table 7:** Lipid classes profile, molecular species of triacylglycerols content regarding their carbon number (CN), and relative composition of phospholipids (g/100 g of fat) in human milk from mothers of hospitalized very preterm infants (*n* = 12).

Lipid Classes	
Triacylglycerols	96.85 (2.15)
Diacylglycerols	2.77 (1.98)
Monoacylglycerols	0.02 (0.01, 0.04)
Free fatty acids + cholesterol	0.31 (0.13, 0.39)
Polar lipids	0.05 (0.05, 0.07)
**Triacylglycerols**	
CN24	0.01 (0.01, 0.01)
CN26	0.08 (0.07, 0.12)
CN28	0.04 (0.01, 0.05)
CN30	0.09 (0.03, 0.11)
CN32	0.09 (0.06, 0.17)
CN34	0.18 (0.06, 0.27)
CN36	0.50 (0.17)
CN38	1.28 (0.34)
CN40	2.07 (0.64)
CN42	3.08 (1.48)
CN44	5.47 (2.05)
CN46	7.85 (2.44)
CN48	10.65 (2.42)
CN50	14.47 (1.90)
CN52	36.76 (6.25)
CN54	15.97 (13.21, 20.12)
**Phospholipids (as % polar lipids)**	
Phosphatidylethanolamine	34.18 (8.33)
Phosphatidylcholine	36.77 (6.46)
Sphingomyelin	29.05 (8.99)

Normally distributed continuous variable: mean (standard deviation). Non-normally distributed continuous variable: median (25th, 75th percentile).

**Table 8:** Fatty acid composition (g/100 g of total fat) in human milk of mothers of hospitalized very preterm infants (*n* = 14).

Fatty Acid (%)	Common Name		Reference Values	
			European [12] ^1^	World [52] ^2^
		**SFAs**		
C6:0	Caproic	0.09 (0.08, 0.09); 0.08 (0.01)	0.08 ± 0.02	0.13 ± 0.47
C8:0	Caprylic	0.21 (0.15, 0.24); 0.20 (0.01)	0.22 ± 0.06	0.21 ± 0.22
C10:0	Capric	1.27 (0.35)	1.44 ± 0.34	1.37 ± 0.86
C12:0	Lauric	5.15 (1.82)	5.46 ± 1.84	5.70 ± 2.81
C14:0	Myristic	5.38 (3.54, 7.55); 5.53 (2.01)	6.19 ± 1.93	6.56 ± 3.05
C15:0	Pentadeccanoic	0.17 (0.13, 0.23); 0.17 (2.01)		
C15:0 ai	C15:0 anteiso	0.02 (0.02, 0.03); 0.02 (0.01)		
C15:0 i	C15:0 iso	0.03 (0.02, 0.05) 0.03 (0.03)		
C16:0	Palmitic	19.64 (2.13)	21.94 ± 2.92	21.5 ± 4.82
C16:0 i	C16:0 iso	0.02 (0.01, 0.02); 0.02 (0.02)		
C17:0	Margaric	0.18 (0.15, 0.21) 0.18 (0.05)		
C17:0 ai	C17:0 anteiso	0.04 (0.03, 0.05); 0.04 (0.03)		
C17:0 i	C17:0 iso	0.27 (0.08)		0.31 ± 0.15
C18:0	Stearic	6.11 (1.01)	6.68 ± 1.59	6.36 ± 2.07
C20:0	Arachidic	0.17 (0.12, 0.24); 0.17 (0.06)	0.17 ± 0.04	0.23 ± 0.17
		**MUFAs**		
C14:1 *cis*-9 (n5)	Myristoleic	0.06 (0.04, 0.11); 0.07 (0.04)		
C16:1 *cis*-9 (n7)	Palmitoleic	1.47 (1.10, 1.66); 1.39 (0.36)	2.21 ± 0.64	2.30 ± 0.92
C17:1	Margaroleic	0.06 (0.03)		
∑ C18:1 *trans*		0.16 (0.10. 0.24); 0.19 (0.12)	0.66 ± 0.35	
C18:1 *cis*-9 (n9)	Oleic	37.33 (6.09)	35.59 ± 4.17	32.60 ± 5.84
C18:1 *cis*-11 (n7)	*Cis*-vaccenic	1.49 (0.24)	2.38 ± 0.53	
C20:1 (n9)	Gondoic	0.78 (0.35, 0.97); 0.79 (0.54)	0.38 ± 0.12	0.46 ± 0.28
		***n*-6 PUFAs**		
**C18:2 (n6)**	**Linoleic**	16.90 (13.97, 18.77) 16.72 (3.16)	14.0 ± 4.95	15.7 ± 7.15
**C20:2 (n6)**	**Eicosadienoic**	0.39 (0.30, 0.55); 0.44 (0.17)	0.26 ± 0.07	0.37 ± 0.19
**C20:3 (n6)**	**Dihomo-γ-linolenic**	0.45 (0.37, 0.60); 0.51 (0.22)	0.31 ± 0.09	0.37 ± 0.18
**C20:4 (n6)**	**Arachidonic**	0.54 (0.20)	0.44 ± 0.12	0.50 ± 0.25
		***n*-3 PUFAs**		
**C18:3 (n3)**	**Linolenic (ALA)**	0.67 (0.52, 0.84); 0.69 (0.25)	0.94 ± 0.55	1.11 ± 1.05
**C22:5 (n3)**	**Docosapentaenoic**	0.08 (0.04, 0.11); 0.10 (0.25)		
**C22:6 (n3)**	**Docosahexaenoic**	0.41 (0.17, 0.49); 0.47 (0.48)	0.34 ± 0.35	0.37 ± 0.31
		***n*-7 PUFAs**		
**C18:2 c9, t11 (n7)**	**Rumenic**	0.04 (0.03, 0.08); 0.06 (0.06)		
		**Fatty Acid**		
**Not identified**		0.23 (0.15, 0.30); 0.25 (0.15)		
**SFAs**		39.06 (35.77, 42.42); 38.91 (4.74)	42.23 ± 5.29	42.2 ±7.73
**MUFAs**		41.31 (6.24); 41.31 (6.24)	41.34 ± 4.48	36.3 ± 6.46
**PUFAs**		19.55 (16.54, 21.69); 19.53 (3.58)	16.43 ± 5.07	21.2 ± 8.18
**SCFAs**		0.09 (0.08, 0.09); 0.08 (0.01)		
**MCFAs (C8-C15)**		11.68 (8.75, 16.67); 12.45 (4.07)		
**LCFAs (C16-C18)**		84.32 (79.77, 88.19); 84.13 (4.30)		
**VLCFAs (C20-C24)**		2.94 (2.55, 3.55); 3.08 (0.73)		
***n*-6 PUFAs**		18.27 (15.34, 20.66); 18.20 (3.28)		17.8 ± 7.51
***n*-3 PUFAs**		1.09 (0.82, 1.43); 1.26 (0.78)		1.88 ± 2.63
***n*-6 PUFAs/*n*-3 PUFAs**		15.19 (11.76, 24.08); 18.65 (10.26)		
**LA/ALA ratio**		20.36 (18.22, 36.38); 28.21 (14.86)		
**ARA/DHA ratio**		1.31 (0.94, 3.35); 2.11 (1.72)	1.68 ± 0.89	

Normally distributed continuous variable: mean (standard deviation). Non-normally distributed continuous variable: median (25th, 75th percentile). The mean standard derivation is shown after the semicolons for comparison with reference values. ^1^ A total of 223 lactating mothers, with a lactation stage of 120 ± 5 days (mean ± standard deviation). ^2^ Mature milk: mean ± standard deviation. Abbreviations: LCFAs—long-chain fatty acids, MCFAs—medium-chain fatty acids, MUFAs—monounsaturated fatty acids, PUFAs—polyunsaturated fatty acids, SCFAs—short-chain fatty acids, SFAs—saturated fatty acids, and VLCFAs—very long-chain fatty acids.

**Table 9:** Macronutrient, vitamins and minerals composition in the human milk of mothers of hospitalized very preterm infants.

Variable ^1^	Unit	Reference ^1^	*n*	Macronutrients
Lipids	g/dL	3.5	15	3.36 (2.27, 3.95)
Carbohydrates	g/dL	6.4	15	7.75 (7.57, 7.96)
Proteins	g/dL	0.94	15	1.36 (1.17, 1.45)
				**Vitamins**
Free thiamin, B1	µg/L	18.5	15	10.70 (7.53, 22.13)
Free riboflavin, B2	µg/L	11.2	15	32.98 (12.33, 71.70)
Nicotinamide, B3	µg/L	275	15	55.73 (35.78. 95.03)
Pantothenic acid, B5	µg/L	2500	15	2334.63 (1795.30, 2634.95)
Pyridoxal, B6	µg/L	96	15	24.43 (18.98, 68.93)
Folic Acid, B9	µg/L		15	19.88 (7.04)
Cobalamin, B12	pM		15	478.08 (456.95, 561.93)
	µg/L	0.5		0.65 (0.62, 0.76)
Retinol	µg/dL		15	47.75 (36.65, 69.25)
	µg/L	530		477.5 (366.5, 692.5)
Ascorbic acid	µg/L		15	4.51 (2.75)
Dehydroascorbic acid	µg/L		15	1.98 (1.07, 2.57)
Vitamin C total *	µg/dL		15	6.27 (4.51, 7.29)
	mg/L	35–90		62.7 (45.1, 72.9)
Vitamin D_3_	pg/mL		15	805.38 (151.17, 4142.30)
	µg/L	0.25–2.00		0.81 (0.15, 0.41)
25(OH)D_3_	pg/mL		15	66.23 (35.05, 171.13)
α-tocopherol	µg/dL		15	415.03 (383.00, 547.35)
	mg/L	4.6		4.15 (3.83, 5.47)
γ-tocopherol	µg/dL		15	40.48 (35.33, 55.85)
	mg/L	0.45		0.40 (0.35, 0.56)
				**Minerals**
Calcium	mg/L	200–300	15	108.75 (63.40, 125.40)
Iodine	µg/L	100–200	15	236.25 (137.08, 338.98)
Phosphorous	mg/L	120–140	15	131.36 (23.45)
Selenium	µg/L	18	15	13.38 (10.65, 16.08)

Normally distributed continuous variable: mean (standard deviation). Non-normally distributed continuous variable: median (25th, 75th percentile). For comparison, the units of our results were converted to those used in the references for the nutrients in human milk. ^1^ Mature milk nutrient concentration references compiled from different experimental studies [14,53,54,55], as well as the European Food Safety Authority NDA panels [56,57,58,59,60,61,62,63,64,65,66,67,68]. * Vitamin C = ascorbic acid + dehydroascorbic acid.

**Table 10:** Coefficient B (95% CI) generated via linear regression with bootstrap sampling for diverse human milk nutrient concentrations according to study group (MHVPI vs. HMD), nutrient intake, and plasma, erythrocyte, and urine status.

HM		MHVPI (Yes) ^1^	Intake		Status	Plasma	Erythrocytes
	*n*	B (95% CI) ^2^		B (95% CI) ^2^		B (95% CI) ^2^	B (95% CI) ^2^
**Lipids**	115	0.150 (−0.613, 0.879)	**Total fat**	0.000 (−0.009, 0.009)	**- ^3^**	-	-
**Carbohydrates**	115	0.019 (−0.142, 0.198)	**Kcal carbohydrates**	0.000 (−0.10, 0.009)	**-**	-	-
**Proteins**	115	**0.169 (0.039, 0.295)**	**Protein**	0.001 (−0.001, 0.003)	**-**	-	-
**Free thiamin, B1**	125	−7.265 (−16.067, 3.325)	**Vitamin B1**	−1.530 (−5.466, 2.697)	**Thiamin, B1**	8.179 (−1.475, 18.926)	-
**Free riboflavin, B2**	125	**−52.42 (−97.48, −7.36)**	**Vitamin B2**	**29.67 (13.99, 45.36)**	**Riboflavin, B2**	0.045 (−0.771, 0.861)	**0.040 (0.001, 0.079)**
**Nicotinamide, B3**	125	2.176 (−28.284, 33.321)	**Vitamin B3**	−0.415 (−1.0127, 0.286)	**Nicotinamide, B3**	0.834 (−1.987, 2.903)	**0.005 (0.002, 0.008)**
**Pantothenic acid, B5**	125	−53.23 (−339.43, 286.15)	**Vitamin B5**	**37.93 (10.25, 55.09)**	**Pantothenic acid, B5**	−0.018 (−0.750, 4.965)	0.616 (−0.366, 1.901)
**Pyridoxal, B6 ^4^**	125	−3.224 (−18.396, 12.437)	**Vitamin B6**	2.475 (−0.820, 5.939)	**Pyridoxamine, B6**	−0.072 (−0.143, 0.002)	−0.006 (−0.022, 0.013)
					**Pyridoxine, B6**	−0.039 (−0.169, 0.091)	-
**Folic acid, B9**	125	−1.345 (−5.680, 3.672)	**Folate + folic acid, B9**	0.000 (−0.002, 0.010)	**Folic acid, B9**	0.023 (−0.304, 1.269)	-
**Cobalamin, B12**	124	0.716 (−41.798, 50.244)	**Vitamin B12**	0.279 (−4.253, 5.961)	**Cobalamin, B12**	**0.133 (0.075, 0.189)**	-
**Cobalamin, B12**	124	−1.092 (−48.401, 52.695)	**Vitamin B12**	0.242 (−4.784, 3.766)	**Holotranscobalamina II**	**0.405 (0.215, 0.597)**	-
**Retinol, A**	123	−890.90 (−2079.43, 83.33)	**Vitamin A**	−0.049 (−0.673, 0.008)	**Retinol, A**	15.91 (−19.51, 70.61)	-
**Ascorbic acid**	124	1.020 (−0.287, 2.431)	**Vitamin C**	0.000 (−0.003, 0.004)	**Ascorbic acid, C**	**0.013 (0.002, 0.022)**	-
**Dehydroascorbic acid**	124	**−0.872 (−1.551, −0.225)**	**Vitamin C**	0.001 (−0.002, 0.004)	**Ascorbic acid, C**	**−0.015 (−0.022, −0.007)**	-
**Vitamin D_3_**	125	1964.72 (−2848.29, 8140.45)	**Vitamin D**	−12.20 (−176.25, 171.06)	**25(OH)D_3_**	**−372.12 (−648.24, −157.44)**	-
**Vitamin D_3_**	125	2443.43 (−2482.43, 8636.93)	**Vitamin D**	−65.07 (−242.03, 117.98)	**1,25(OH)D_2_**	**−9.659 (−16.174, −5.283)**	-
**Vitamin D_3_**	111	3182.52 (−2132.89, 10027.89)	**Vitamin D**	22.61 (−125.04, 164.55)	**D_3_**	4.545 (−0.307, 24.944)	-
**25(OH)D_3_**	125	**68.29 (6.34, 115.19)**	**Vitamin D**	1.814 (−1.108, 5.315)	**25(OH)D_3_**	**6.095 (2.110, 10.410)**	-
**25(OH)D_3_**	125	**60.44 (0.24, 107.93)**	**Vitamin D**	2.681 (−0.239, 6.229)	**1,25(OH)D_2_**	**0.159 (0.094, 0.248)**	-
**α-tocopherol**	122	15.52 (−64.25, 105.57)	**Vitamin E**	2.632 (−1.151, 6.585)	**α-tocopherol**	−0.007 (−0.081, 0.074)	-
**α-tocopherol**	120	16.19 (−66.60, 116.12)	**Vitamin E**	2.556 (−1.235, 6.794)	**γ-tocopherol**	−0.184 (−1.926, 1.117)	-
**γ-tocopherol**	122	−4.444 (−14.268, 5.982)	**Vitamin E**	−0.188 (−0.600, 0.284)	**α-tocopherol**	**−0.014 (−0.023, −0.006)**	-
**γ-tocopherol**	120	−10.049 (−20.045, 1.226)	**Vitamin E**	−0.115 (−0.588, 0.379)	**γ-tocopherol**	0.180 (−0.081, 0.370)	-
**Calcium**	125	−9.579 (−38.346, 20.525)	**Calcium**	−0.014 (−0.039, 0.012)	**Calcium ^5^**	0.103 (0.014, 0.192)	-
**Calcium**	125	−7.905 (−34.523, 16.143)	**Calcium**	−0.013 (−0.036, 0.011)	**Calcium/Cr ^5^**	14.01 (−37.34, 176.13)	-
**Iodine**	125	**73.96 (9.70, 142.75)**	**Iodine**	**0.268 (0.127, 0.417)**	**Iodine ^5^**	0.093 (−0.175, 0.319)	-
**Iodine**	125	**67.66 (7.86, 129.36)**	**Iodine**	**0.255 (0.112, 0.402)**	**Iodine/Cr ^5^**	145.12 (−152.47, 485.33)	-
**Phosphorous**	125	−1.395 (−18.612, 16.850)	**Phosphorous**	−0.006 (−0.020, 0.007)	**Phosphorous ^5^**	**−0.009 (−0.017, −0.001)**	-
**Phosphorous**	125	−0.0565 (−18.341, 13.369)	**Phosphorous**	−0.004 (−0.018, 0.009)	**Phosphorous/Cr ^5^**	**−16.817 (−30.660, −2.322)**	-
**Selenium**	125	**5.776 (2.498, 8.747)**	**Selenium**	0.011 (−0.010, 0.033)	**Selenium ^5^**	-	-
**SFAs**	119	1.145 (−1.489, 4.264)	**SFAs**	**0.095 (0.010, 0.197)**	**SFAs**	**0.437 (0.025, 0.914)**	0.115 (−0.131, 0.387)
**MUFAs**	119	−3.160 (−6.780–0.381)	**MUFAs**	0.046 (−0.033, 0.121)	**MUFAs**	**0.402 (0.122, 0.768)**	-
**MUFAs**	120	−2.444 (−6.013, 0.882)	**MUFAs**	0.037 (−0.042, 0.113)	**MUFAs**	-	**0.777 (0.131, 1.378)**
**PUFAs**	119	2.111 (−0.073, 4.455)	**PUFAs**	0.109 (−0.005, 0.248)	**PUFAs**	0.047 (−0.132, 0.204)	−0.077 (−0.290, 0.124)
***n*-6 PUFAs**	119	1.671 (−0.403, 3.570)	***n*-6 PUFAs**	0.106 (−0.019, 0.258)	***n*-6 PUFAs**	0.071 (−0.096, 0.235)	-
***n*-6 PUFAs**	120	1.735 (−0.376, 3.799)	***n*-6 PUFAs**	0.107 (−0.026, 0.266)	***n*-6 PUFAs**	-	0.040 (−0.191, 0.286)
***n*-3 PUFAs**	119	**0.396 (0.039, 0.830)**	***n*-3 PUFAs**	0.055 (−0.022, 0.177)	***n*-3 PUFAs**	**0.094 (0.023, 0.209)**	-
***n*-3 PUFAs**	120	**0.349 (0.009, 0.797)**	***n*-3 PUFAs**	0.057 (−0.019, 0.171)	***n*-3 PUFAs**	-	**0.059 (0.018, 0.100)**
***n*-6 PUFAs/*n*-3 PUFAs**	119	−2.412 (−8.325, 4.135)	***n*-6 PUFAs/*n*-3 PUFAs**	**0.783 (0.088, 1.564)**	***n*-6 PUFAs/*n*-3 PUFAs**	**0.038 (0.007, 0.081)**	-
***n*-6 PUFAs/*n*-3 PUFAs**	120	−2.210 (−8.183, 4.472)	***n*-6 PUFAs/*n*-3 PUFAs**	**0.822 (0.173, 1.587)**	***n*-6 PUFAs/*n*-3 PUFAs**	-	**0.396 (0.067, 0.849)**
**MCFAs (C8-C15)**	119	0.575 (−1.819, 3.355)	**MCFAs (C8-C15)**	-	**MCFAs (C8-C15)**	2.887 (−1.849, 8.796)	-
**MCFAs (C8-C15)**	120	0.976 (−1.632, 4.044)	**MCFAs (C8-C15)**	-	**MCFAs (C8-C15)**	-	4.898 (−16.109, 26.778)
**LCFAs (C16-C18)**	119	−1.684 (−4.359, 0.904)	**LCFAs (C16-C18)**	-	**LCFAs (C16-C18)**	0.126 (−0.078, 0.312)	-
**LCFAs (C16-C18)**	119	−1.597 (−4.092, 0.664)	**LCFAs (C16-C18)**	-	**LCFAs (C16-C18)**	-	−0.082 (−0.225, 0.054)
**VLCFAs (C20-C24)**	119	**0.675 (0.267, 1.097)**	**VLCFAs (C20-C24)**	-	**VLCFAs (C20-C24)**	0.017 (−0.027, 0.065)	-
**VLCFAs (C20-C24)**	120	**0.659 (0.248, 1.073)**	**VLCFAs (C20-C24)**	-	**VLCFAs (C20-C24)**	-	−0.029 (−0.068, 0.009)
**C14:0**	119	0.293 (−0.886, 1.695)	**C14:0**	−0.016 (−0.312, 0.305))	**C14:0**	1.976 (−0.694, 5.146)	-
**C14:0**	120	0.511 (−0.845, 2.113)	**C14:0**	0.024 (−0.241, 0.381)	**C14:0**	-	0.699 (−13.512, 13.482)
**C15:0**	19	−0.025 (−0.067, 0.015)	**C15:0**	-	**C15:0**	**2.191 (1.353, 3.051)**	-
**C16:0**	119	0.007 (−1.392, 1.273)	**C16:0**	**0.117 (0.036. 0.181)**	**C16:0**	**0.296 (0.032, 0.550)**	−0.051 (−0.276, 0.183)
**C18:0**	119	0.213 (−0.487, 0.944)	**C18:0**	**0.107 (0.017, 0.195)**	**C18:0**	**0.401 (0.042, 0.855)**	−0.037 (−0.188, 0.113)
**C16:1 *cis*-9**	119	**−0.348 (−0.595, −0.119)**	**C16:1**	0.099 (−0.047, 0.271)	**C16:1**	**0.568 (0.281, 1.016)**	-
**C17:1**	120	−0.014 (−0.028, 0.001)	**C17:1**	-	**C17:1**	-	**−0.044 (−0.083, −0.008)**
**C18:1 *cis*-9**	119	−2.630 (−5.951, 0.600)	**C18:1**	0.050 (−0.029, 0.120)	**C18:1 *cis*-9**	**0.436 (0.185, 0.784)**	-
**C18:1 *cis*-9**	120	−2.096 (−5.397, 1.279)	**C18:1**	0.043 (−0.037, 0.121)	**C18:1 *cis*-9**	-	**0.672 (0.057, 1.285)**
**C18:1 *cis*-11**	119	**−0.202 (−0.360, −0.046)**	**C18:1**	0.001 (−0.004, 0.006)	**C18:1 *cis*-11**	**0.374 (0.115, 0.662)**	-
**C18:1 *cis*-11**	120	**−0.196 (−0.355, −0.032)**	**C18:1**	0.000 (−0.004, 0.005)	**C18:1 *cis*-11**	-	0.751 (−0.002, 1.471)
**C18:2**	119	1.221 (−0.830, 3.146)	**C18:2**	0.103 (−0.024, 0.250)	**C18:2**	0.089 (−0.014, 0.190)	-
**C18:2**	120	0.977 (−1.063, 2.937)	**C18:2**	0.096 (−0.032, 0.239)	**C18:2**	-	0.415 (−0.045, 0.820)
**C20:3**	119	**0.134 (0.036, 0.232)**	**C20:3**	-	**C20:3**	**0.069 (0.022, 0.129)**	-
**C20:3**	120	**0.177 (0.047, 0.304)**	**C20:3**	-	**C20:3**	-	−0.033 (−0.101, 0.028)
**C20:4**	119	0.042 (−0.042, 0.121)	**C20:4**	-	**C20:4**	**0.031 (0.022, 0.039)**	-
**C20:4**	120	0.004 (−0.105, 0.122)	**C20:4**	-	**C20:4**	-	0.007 (−0.027, 0.039)
**C18:3**	120	**0.243 (0.102, 0.396)**	**C18:3**	0.006 (−0.035, 0.068)	**C18:3**	-	-
**C22:5**	120	0.020 (−0.016, 0.061)	**C22:5**	**0.596 (0.380, 0.795)**	**C22:5**	-	0.008 (−0.015, 0.028)
**C22:6**	119	0.074 (−0.108, 0.303)	**C22:6**	**0.408 (0.271, 0.564)**	**C22:6**	**0.131 (0.051, 0.225)**	-
**C22:6**	120	0.044 (−0.129, 0.286)	**C22:6**	**0.411 (0.280, 0.569)**	**C22:6**	-	**0.057 (0.029, 0.084)**

^1^ Reference category: (Coefficient B = 0) = HMD. ^2^ B (95% CI): generated via multivariate linear regression and 5000 bias-corrected and accelerated bootstrap sample. ^3^ Variables not introduced in the linear regression, either because they were not assessed or because the plasma and erythrocyte status showed co-correlation and were, therefore, assessed in a different regression analysis. ^4^ Two vitamers of vitamin B6 were measured in plasma status. Both were introduced in the same regression analysis. ^5^ Measured in urine. Values in bold: ***p*** < **0.05**. Abbreviation: CI—confidence interval, HM—human milk, HMDs—human milk donors, MHVPIs—mothers of hospitalized very preterm infants, LCFAs—long-chain fatty acids, MCFAs—medium-chain fatty acids, MUFAs—monounsaturated fatty acids, PUFAs—polyunsaturated fatty acids, SCFAs—short-chain fatty acids, SFAs—saturated fatty acids, VLCFAs—very long-chain fatty acids.

## Data Availability

The data presented in this study are available on request from the corresponding author. The data are not publicly available due to privacy issues.

## Supplementary Materials

The following supporting information can be downloaded at: https://www.mdpi.com/article/10.3390/nu17111932/s1, Table S1: Baseline characteristics according to study group: mothers of hospitalized very preterm infants (MHVPI) (*n* = 15) and human milk donors (HMD) (*n* = 110); Table S2: Characteristics of the infant and of lactation according to study group: mothers of hospitalized very preterm infants (MHVPI) (*n* = 15) and human milk donors (HMD) (*n* = 110); Table S3: Intake of pharmacological dietary supplements during pregnancy and lactation according to study group: mothers of hospitalized very preterm infants (MHVPI) (*n* = 15) and human milk donors (HMD) (*n* = 110); Table S4: Daily nutrients intake determined according to the 5-day dietary record according to study group: mothers of hospitalized very preterm infants (MHVPI) (*n* = 15) and human milk donors (HMD) (*n* = 110); Table S5: The prevalence of inadequate nutrient intake, regarding the harmonized average requirement (H-AR) proposed by Allen et al. [14], recommended daily intakes from Ortega et al. [35], and acceptable macronutrient distribution ranges from the Institute of Medicine [36] according to study group: mothers of hospitalized very preterm infants (MHVPI) (*n* = 15) and human milk donors (HMD) (*n* = 110); Table S6: Daily number of food servings, and intake of supplements and iodized salt determined using the 5-day dietary record according to study group: mothers of hospitalized very preterm infants (MHVPI) (*n* = 15) and human milk donors (HMD) (*n* = 110); Table S7: Number of food portions determined using the food consumption frequency questionnaire according to study group: mothers of hospitalized very preterm infants (MHVPI) and human milk donors (HMD); Table S8: Fatty acid composition (g/100 g of total fat) in the erythrocytes and plasma samples according to study group: mothers of hospitalized very preterm infants (MHVPI) (*n* = 14) and human milk donors (HMD) (*n* = 110); Table S9: Concentrations of nutrients and biochemical determinations in the erythrocytes, plasma, and urine according to study group: mothers of hospitalized very preterm infants (MHVPI) and human milk donors (HMD); Table S10: Lipid classes profile, molecular species of triacylglycerol content regarding their carbon number (CN), and relative composition of phospholipids in human milk according to study group: mothers of hospitalized very preterm infants (MHVPI) (*n* = 12) and human milk donors (HMD) (*n* = 20); Table S11: Fatty acid composition (g/100 g of total fat) in human milk according to study group: mothers of hospitalized very preterm infants (MHVPI) (*n* = 14) and human milk donors (HMD) (*n* = 106); Table S12: Macronutrient, vitamin, and mineral composition in the human milk according to study group: mothers of hospitalized very preterm infants (MHVPI) and human milk donors (HMD) [109,110,111].

## Abbreviations

The following abbreviations are used in this manuscript:

BFHI	Baby-friendly Hospital Initiative
BMI	Body mass index 1
CI	Confidence intervals
CN	Carbon numbers
DHA	Docosahexaenoic acid
DMA	Dimethylacetal
EFSA	European Food Safety Authority
EPA	Eicosapentaenoic acid
FA	Fatty acid
FAME	Fatty acid methyl ester
H-AR	Harmonized Average Requirement
HEI	Healthy eating index
HDL	High-density lipoprotein
HM	Human milk
HMDs	Human milk donors
IOM	Institute of Medicine (National Academy of Medicine (NAM))
Kcal	Kilocalories
LDL	Low-density lipoprotein
MHVPIs	Mothers of hospitalized very preterm infants
MUFA	Monounsaturated fatty acid
NICU	Neonatal intensive care unit
PUFA	Polyunsaturated fatty acid
RHMB	Regional Human Milk Bank “Aladina MGU”
TAG	Triacylglycerol
SCFA	Short-chain fatty acid
SFA	Saturated fatty acid
