# Supplementary material for: Nutrient Intake and Plasma and Erythrocyte Content Among Lactating Mothers of Hospitalized Very Preterm Infants: Associations with Human Milk Composition
Source: Nutrients. 2025 Jun 4;17(11):1932. doi: 10.3390/nu17111932 (PMC12158112; doi:10.3390/nu17111932)
Supplement: Supplementary file 1 [file nutrients-17-01932-s001.zip › nutrients-3599381-supplementary.pdf]

Table S1. Baseline characteristics according to study group: Mothers of hospitalized very-preterm infants (MHVPI) (*n*=15) and human milk donors (HMD) (*n*=110).

Characteristics	MHVPI	HMD	p-value
**Age (years)**	35.7 (6.6); 26.1-51.5	35.9 (4.2); 26.3-47.0	0.864
**Weight (kg)**	70.2 (59.6, 77.0); 44.4-88.4	60.5 (55.2, 70.6); 46.0-122.0	0.115
**Height (cm)**	163.9 (8.7); 151.3-176.7)	164.0 (6.3); 151.2-181.0	0.966
**Pre-pregnancy BMI (kg/m2)**	24.5 (20.1, 29.8); 17.6-36.2	22.1 (50.6, 24.8); 17.7-44.2	0.371
**Pre-pregnancy BMI (kg/m2) category**			
Underweight (<18.5)	1 (6.7)	3 (2.7)	0.061
Normal (18.5–24.9)	7 (46.7)	83 (75.5)	
Overweight (25–29.9)	4 (26.7)	14 (12.7)	
Obese (≥30)	3 (20.0)	10 (9.1)	
**Current BMI (kg/m2)**	24.7 (22.5,29.1); 18.1-35.7	22.9 (21.1, 25.0); 16.7-42.8	0.219
**Current BMI (kg/m2) category**	1 (6.7)	3 (2.7)	0.109
Underweight (<18.5)	7 (46.7)	80 (72.7)	
Normal (18.5–24.9)	4 (26.7)	14 (12.7)	
Overweight (25–29.9)	3 (20.0)	13 (11.8)	
Obese (≥30)			
**Gestational weight gain (kg)**	7.0 (3.5, 10.0); 0.0-12.0	12.0 (9.0, 14.6); -5.0-30.0	**0.000**
**Postpartum weight retention (kg)**	1.4 (-0.6, 4.4); -8.0-10.2	0.95 (-0.63, 2.45); -12.0-17.0	0.414
**Number of children[1]**			
1	7 (46.7)	60 (55.0)	0.779
2	6 (40.0)	38 (34.9)	
≥3	2 (13.3)	11 (10.1)	
**Country of origin: Spain**	9 (60.2)	99 (90.0)	**0.006**
**Education level**			
Secondary studies	1 (6.7)	2 (1.8)	0.168
Technical studies	3 (20.0)	13 (11.8)	
University studies	11 (73.3)	95 (86.4)	
**Currently working (yes)**	2 (13.3)	50 (45.5)	**0.024**
**Physical activity**			
Sedentary	6 (40.0)	25 (22.7)	0.354
Low active	7 (46.7)	58 (52.7)	
Active/very active	2 (13.3)	27 (24.5)	
**Tobacco before pregnancy (yes)**	3 (20.0)	22 (20.0)	1.000
**Tobacco during pregnancy (yes)**	0 (0)	3 (2.7)	1.000
**Active smoking actually (yes)**	1 (6.7)	1 (0.9)	0.226
**Passive smoking (yes)**	3 (20.0)	23 (20.9)	1.000
**Alcohol consumption**			
Prior to pregnancy (yes)	7 (46.7)	55 (50.0)	1.000
During pregnancy (yes)	0 (0.0)	1 (0.9)	1.000
Currently (yes)	0 (0.0)	4 (3.6)	1.000
**Season during the study**			
Spring	2 (13.3)	28 (25.5)	0.106
Summer	2 (13.3)	16 (14.5)	
Autumn	3 (20.0)	41 (37.3)	
Winter	8 (53.3)	25 (22.7)	

Normally distributed continuous variable: mean (standard deviation).

Non-normally distributed continuous variable: median (25th, 75th percentile).

Qualitative variable: absolute (*n*) and relative frequencies (%).

Ranges for quantitative variables are shown after the semicolons.

[1] Donor group with an n = 109 due to stillbirth.

Abbreviations: BMI – body mass index.

Values in bold: $p < 0.05$.

Table S2. Characteristics of the infant and of lactation according to study group: Mothers of hospitalized very-preterm infants (MHVPI) (*n*=15) and human milk donors (HMD) (*n*=110).

Characteristics	MHVPI	HMD	p-value
**Sex (Girl)**	13 (65.0)	57 (51.8)	0.334
**Twin pregnancy** [1]	6 (40.0)	2 (1.8)	**<0.001**
**Gestational age (weeks)**	26 (24, 27); 23-30	39 (38, 40); 22-42	**<0.001**
**Birth weight (g)** [2]	735.0 (642.5, 990.0); 535-1750	3242.5 (2847.5, 3481.3); 450-4640	**<0.001**
**Birth weight percentiles** [2,3]			
<25	5 (25.0)	9 (8.2)	**0.005**
25-75	10 (50.0)	91 (82.7)	
>75	5 (25.0)	10 (9.1)	
**Currently weight (g)** [4]	2127.5 (1450.0, 2700.0); 725-2950	7800.0 (6440.0, 9130.0); 3150-18000	**<0.001**
**Currently weight percentiles** [4,5]			
<15	10 (50.0)	14 (12.8)	**0.001**
15-85	10 (50.0)	93 (85.3)	
>85	0 (0.0)	2 (1.8)	
**Breastfeeding duration (months)**	1.7 (1.1, 2.5); 0.7-3.2	7.0 (4.8, 11.9); 0.6-49.4	**<0.001**
**Breastfeeding duration categories**			
0-5 months	15 (100.0)	46 (41.8)	**0.001**
6-12 months	0 (0.0)	37 (33.6)	
12-23 months	0 (0.0)	22 (20.0)	
24-50 months	0 (0.0)	5 (4.5)	
**Type of lactation**			
Exclusive	15 (100.0)	48 (43.6)	**0.001**
Partial [6]	0 (0.0)	61 (55.59)	
Missing data	0 (0.0)	1 (0.9)	
**Sum of child breastfeeding times and pump sessions per day**			
<5	0 (0.0)	12 (10.9)	0.271
5-10	12 (80.0)	63 (57.3)	
>10	3 (20.0)	32 (29.1)	
Missing data	0 (0.0)	3 (2.7)	
**Tandem breastfeeding (yes)**	4 (26.7)	5 (4.5)	0.120
**Milk expression type** [7]			
Manual	0 (0.0)	7 (6.4)	0.597
Mechanical breast pump	1 (6.7)	12 (10.9)	0.708
Simple electric breast pump	8 (53.3)	81 (73.6)	0.130
Double electric breast pump	9 (60.0)	15 (13.6)	**<0.001**

Normally distributed continuous variable: mean (standard deviation).

Non-normally distributed continuous variable: median (25th, 75th percentile).

Qualitative variable: absolute (*n*) and relative frequencies (%).

Ranges for quantitative variables are shown after the semicolons.

[1] One of the MHVPI from a twin pregnancy lost one of the children before giving birth.

[2] *n*=20, regarding MHVPI group because of twin birth.

[3] Olsen intrauterine growth curves [109].

[4] One lose in the HMD group due to stillbirth and two loses in the MHVPI group due to still birth and death of one of the twins after birth.

[5] World Health Organization (WHO)'s child growth standards [110].

[6] Complementary food introduction.

[7] Categories do not exclude each other.

Values in bold: p < 0.05.

Table S3. Intake of pharmacological dietary supplements during pregnancy and lactation according to study group: Mothers of hospitalized very-preterm infants (MHVPI) (*n*=15) and human milk donors (HMD) (*n*=110).

	**Frequency**	***n (%)***		**Daily dose** (Median (25th percentile, 75th percentile); Range)		
	**MHVPI**	**HMD**	**p-value**	**MHVPI**	**HMD**	**p-value**
**Vitamin A, μg**						
**Pregnancy**	2 (13.3)	16 (14.5)	1.000	700.0 (-); 700.0-700.0	700.0 (349.8, 700.0); 23.0-800.0	0.497
**Lactation**	8 (53.3)	55 (50.0)	1.000			
Previously [1, 2]	1 (12.5)	14 (25.5)	0.667	800.0 (-); 800.0-800.0	800.0 (725.0, 800.0); 333.0-1000.0	1.000
Currently [1,3]	7 (87.5)	41 (74.5)		800.0 (400.0, 800.0); 400.0-1000.0	800.0 (400.0, 800.0); 160.0-1000.0	0.876
**Vitamin D, μg**						
**Pregnancy**	7 (46.7)	59 (53.6)	0.784	10.0 (5.0, 15.0); 5.0-15.0	10.0 (5.0, 10.0); 3.8-30.0	0.486
**Lactation**	8 (53.3)	63 (57.3)	0.788			
Previously	1 (12.5)	15 (23.8)	0.673	5 (-); 5.0-5.0	5.0 (5.0, 5.0); 2.5-10.0	1.000
Currently	7 (87.5)	48 (76.2)		5.0 (2.5, 5.0); 2.5-5.0	5.0 (3.9, 5.0); 1.0-25.0	0.514
**Vitamin E, mg**						
**Pregnancy**	3 (20.0)	28 (35.5)	0.760	12.0 (5.0, -); 5.0-12.0	12.0 (12.0, 12.0); 1.8-15.0)	0.415
**Lactation**	8 (53.3)	60 (54.5)	1.000			
Previously	1 (12.5)	15 (25.0)	0.670	12.0 (-); 12.0-12.0	12.0 (12.0, 12.0); 6.0-15.0	1.000
Currently	7 (87.5)	45 (27.0)		12.0 (6.0, 12.0); 6.0-15.0	12.0 (8.4, 12.0); 2.4-16.0	0.712
**Vitamin C, mg**						
**Pregnancy**	7 (46.7)	57 (51.8)	0.787	40.0 (40.0, 80.0); 40.0-80.0	60.0 (40.0, 80.0); 12.0-180.0	0.540
**Lactation**	8 (53.3)	62 (56.4)	1.000			
Previously	1 (12.5)	16 (25.8)	0.669	80.0 (-); 80.0-80.0	80.0 (80.0, 80.0); 40.0-110.0	1.000
Currently	7 (87.5)	46 (74.2)		80.0 (40.0, 80.0); 40.0-100.0	80.0 (56.5, 81.3); 16.0-125.0	0.687
**Vitamin B1, mg**						
**Pregnancy**	7 (46.7)	57 (51.8)	0.787	1.1 (-); 1.1-1.1	1.1 (1.1, 1.1); 0.6-1.5	0.572
**Lactation**	8 (53.3)	61 (55.5)	1.000			
Previously	1 (12.5)	16 (26.2)	0.669	1.1 (-); 1.1-1.1	1.1 (1.1, 1.1); 0.6-1.2	1.000
Currently	7 (87.5	45 (73.8)		1.1 (0.8, 1.1); 0.6-1.1	1.1 (0.7, 1.1); 0.2-1.4	0.835
**Vitamin B2, mg**						
**Pregnancy**	7 (46.7)	57 (51.8)	0.787	1.4 (-); 1.4-1.4	1.4 (1.4, 1.4); 0.5-2.5	0.558
**Lactation**	8 (53.3)	61 (55.5)	1.000			
Previously	1 (12.5)	16 (26.2)	0.669	1.4 (-); 1.4-1.4	1.4 (1.4, 1.4); 0.7-1.6	1.000
Currently	7 (87.5	45 (73.8)		1.4 (1.1, 1.4); 0.7-1.6	1.4 (0.8, 1.4); 0.3-2.5	0.886
**Vitamin B3, mg**						
**Pregnancy**	7 (46.7)	57 (51.8)	0.787	16.0 (-); 16.0-16.0	16.0 (16.0, 16.0); 4.0-20.0	0.918
**Lactation**	8 (53.3)	61 (55.5)	1.000			
Previously	1 (12.5)	16 (26.2)	0.669	16.0 (-); 16.0-16.0	16.0 (16.0, 16.0); 8.0-16.0	1.000
Currently	7 (87.5	45 (73.8)		16.0 (8.0, 16.0); 8.0-16.0	16.0 (8.0, 16.0); 3.2-18.0	0.993
**Vitamin B5, mg**						
**Pregnancy**	7 (46.7)	57 (51.8)	0.787	6.0 (-); 6.0-6.0	6.0 (6.0, 6.0); 1.0-10.0	0.611
**Lactation**	8 (53.3)	61 (55.5)	1.000			
Previously	1 (12.5)	16 (26.2)	0.669	6.0 (-); 6.0-6.0	6.0 (6.0, 6.0); 3.0-6.0	1.000
Currently	7 (87.5	45 (73.8)		6.0 (3.0, 6.0); 3.0-6.0	6.0 (3.1, 6.0); 1.0-6.0	1.000
**Vitamin B6, mg**						
**Pregnancy**	8 (53.3)	57 (51.8)	1.000	1.4 (1.4, 1.4); 1.4-5.0	1.4 (1.4, 1.4); 0.7-2.2	0.699
**Lactation**	8 (53.3)	61 (55.5)	1.000			
Previously	1 (12.5)	16 (26.2)	0.669	1.4 (-); 1.4-1.4	1.4 (1.4, 1.4); 0.7-2.2	1.000
Currently	7 (87.5	45 (73.8)		1.4 (0.7, 1.4); 0.7-2.0	1.4 (0.9, 1.4); 0.3-2.0	0.824
**Vitamin B8, μg**						
**Pregnancy**	7 (46.7)	57 (51.8)	0.787	50.0 (-); 50.0-50.0	50.0 (50.0, 50.5); 25-150	0.585
**Lactation**	8 (53.3)	61 (55.5)	1.000			
Previously	1 (12.5)	16 (26.2)	0.669	50.0 (-); 50.0-50.0	50.0 (50.0,50.0); 25-60	1.000
Currently	7 (87.5	45 (73.8)		50.0 (25.0, 50.0); 25-50	50.0 (39.5, 50.0); 10-150	0.873
**Vitamin B9, μg**						

	Frequency	*n (%)*		**Daily dose** (Median (25th percentile, 75th percentile); Range)		
	**MHVPI**	**HMD**	**p-value**	**MHVPI**	**HMD**	**p-value**
**Pregnancy**	14 (93.3)	106 (96.4)	1.000	400.0 (400.0, 1305.3); 400.0-4233.0	400.0 (400.0, 400.0); 162.0-6200.0	**0.008**
**Lactation**	13 (86.7)	91 (82.7)	0.746			
Previously [1]	0.(0.0)	22 (24.4)	0.065	-	200.0 (200.0, 400.0); 100-400	-
Currently [2]	13 (100.0)	68 (75.6)		300.0 (200.0, 400.0); 100.0-400.0	300.0 (200.0, 400.0); 2.0-5200.0	0.967
**Vitamin B12, μg**						
**Pregnancy**	14 (93.3)	103 (93.6)	1.000	2.3 (2.0, 2.5); 2.0-2.5	2.3 (2.0, 2.5); 0.4-288.0	0.807
**Lactation**	13 (86.7)	90 (81.8)	0.740			
Previously	0 (0.0)	22 (24.2)	0.065	-	2.5 (2.0, 2.5); 1.0-3.5	-
Currently	13 (100.0)	69 (75.8)		2.0 (2.0, 2.5); 1.3-2.5	2.0(2.0, 2.5); 0.4-288.0	0.714
**Calcium, mg**						
**Pregnancy**	0 (0.0)	5 (4.5)	0.631	-	150.0 (18.0, 250.0); 12.0-300.0	-
**Lactation**	7 (46.7)	45 (40.9)	0.782			
Previously	1 (14.3)	12 (36.7)	0.664	200 (-); 200-200	200.0 (200.0, 200.0); 24-245	1.000
Currently	6 (85.7)	33 (73.3)		200.0 (100.0, 200.0); 100.0-200.0	200.0 (100.0, 200.0); 40.0-500.0	0.982
**Iodine, μg**						
**Pregnancy**	14 (93.3)	105 (95.5)	1.000	200.0 (200.0, 200.0); 150.0-200.0	200.0 (200.0, 200.0); 46.5-400.0	0.672
**Lactation**	13 (86.7)	94 (85.5)	1.000			
Previously	0 (0.0)	21 (22.3)	0.068	-	200.0 (200.0, 200.0); 100.0-200.0	-
Currently	13 (100.0)	73 (77.7)		200.0 (200.0, 200.0); 100.0-200.0	200.0 (200.0, 200.0); 46.0-300.0	0.968
**Iron, mg**						
**Pregnancy**	8 (53.3)	80 (72.7)	0.139	30.0 (17.5, 41.3); 14.0-80.0	28.0 (28.0, 80.0); 6.5-108.0	0.631
**Lactation**	10 (66.7)	78 (70.9)	0.767			
Previously	1 (10.0)	27 (34.6)	0.159	80 (-); 80.0-80.0	15.0 (14.0, 80.0); 7.0-105.0	0.429
Currently	9 (90.0)	51 (65.4)		14.0 (14.0, 30.5); 7.0-114.0	14.0 (14.0, 33.0); 3.0-114.0	0.446
**Selenium, μg**						
**Pregnancy**	7 (46.7)	49 (44.5)	1.000	55.0 (-); 55.0-55.0	55.05 (55.0, 55.0) 27.0-60.0	0.925
**Lactation**	8 (53.3)	56 (50.9)	1.000			
Previously	1 (12.5)	15 (26.8)	0.667	20 (-); 20.0-20.0)	20.0 (20.0, 30.0); 10.0-55.0	1.000
Currently	7 (87.5)	41 (73.2)		20.0 (15.0, 55.0); 10.0-55.0	20.0 (12.5, 55.0); 4.0-60.0	0.506
**Zinc, mg**						
**Pregnancy**	7 (46.7)	52 (47.3)	1.000	10.0 (-); 10.0-10.0	10.0 (10.0, 10.0); 4.3-15.0	0.949
**Lactation**	8 (53.3)	58 (52.7)	1.000			
Previously	1 (12.5)	16 (27.6)	0.668	10.0 (-); 10.0-10.0	20.0 (10.0, 10.0); 5.0-10.0	1.000
Currently	7 (87.5)	42 (72.4)		10.50 (5.0, 10.0); 5.0-10.0	10.0 (5.75, 10.0); 2.0-10.0	1.000
**Omega 3, g**						
**Pregnancy**	7 (46.7)	57 (51.8)	0.787	0.20 (0.16, 0.20); 0.16-0.22	0.20 (0.20, 0.21); 0.14-0.95	0.416
**Lactation**	8 (53.3)	58 (52.7)	1.000			
Previously	1 (12.5)	15 (25.9)	0.668	0.24 8-9; 0.24-0.24	0.24 (0.20, 0.24); 0.08-0.25	1.000
Currently	7 (87.5)	43 (74.1)		0.24 (0.12, 0.24); 0.12-0.24	0.24 (0.16, 0.24); 0.12-0.95	0.734

[1] % depends on the frequency of the intake of pharmacological dietary supplements during lactation.

[2] Previously: at the time of the study, women had stopped taking supplements during lactation.

[3] Currently: at the time of the study, women still took supplements.

Values in bold: $p < 0.05$.

Table S4. Daily nutrients intake determined according to the 5-day dietary record according to study group: Mothers of hospitalized very-preterm infants (MHVPI) (*n*=15) and human milk donors (HMD) (*n*=110).

	MHVPI	HMD	p-value
Energy (Kcal)	2272.6 (1893.8, 2734.6)	2302.7 (2028.9, 2491,85)	0.872
Protein (g)	93.7 (72.4, 105.6)	93.2 (82.0, 104.0)	0.902
Total fat (g)	104.0 (79.1, 113.5)	101.3 (85.5, 118.8)	0.990
Saturated fat (g)	31.1 (26.9, 39.7)	32.1 (27.3, 42.8)	0.806
Monounsaturated fat (g)	40.4 (30.4, 48.0)	42.3 (35.1, 50.2)	0.656
Polyunsaturated fat (g)	18.8 (12.4, 20.3)	15.2 (12.0, 19.1)	0.331
PUFAs/SFAs	0.59 (0.50, 0.72)	0.51 (0.40, 0.67)	0.162
(PUFAs + MUFAs)/SFAs	2.0 (1.7, 2.2)	1.9 (1.6, 2.3)	0.873
Kcal from carbohydrate (%)	42.8 (4.2)	44.2 (5.9)	0.391
Kcal from protein (%)	17.6 (15.7, 18.5)	16.2 (15.0, 17.7)	0.214
Kcal from fat (%)	39.1 (6.0)	39.9 (4.2)	0.610
Kcal from saturated fat (%)	12.6 (2.5)	12.7 (1.7)	0.847
Kcal from polyunsaturated fat (%)	6.7 (5.9, 8.3)	6.1 (5.1, 7.5)	0.102
Kcal from monounsaturated fat (%)	15.8 (14.2, 18.9)	16.1 (14.5, 19.1)	0.621
Kcal from n-3 fatty acids (%)	0.95 (0.82, 1.22)	0.78 (0.65, 1.01)	**0.016**
n-6 PUFAs (g)	15.6 (9.8, 18.3)	12.6 (9.6, 16.6)	0.359
n-3 PUFAs (g)	2.2 (1.7, 3.1)	1.9 (1.5, 2.6)	0.153
n-6/n-3 PUFAs	7.3 (6.2, 8.9)	7.1 (5.8, 8.9)	0.806
Myristic acid (C14:0) (g)	2.9 (1.9, 3.6)	2.7 (2.0, 3.6)	0.686
Palmitic acid (C16:0)(g)	15.4 (13.6, 20.0)	16.1 (12.8, 19.0)	0.675
Palmitoleic acid (C16:1 n7) (g)	1.5 (0.5)	1.6 (0.5)	0.546
Stearic acid (C18:0) (g)	6.7 (5.1, 8.9)	6.9 (5.5, 8.3)	0.987
Oleic acid (C18:1n9c) (g)	38.3 (27.5, 44.5)	39.3 (31.8, 46.6)	0.687
Linoleic acid (C18:2n6c) (g)	15.5 (9.7, 18.2)	12.4 (9.5, 16.5)	0.355
Linolenic acid (C18:3n3) (g)	1.7 (1.2, 2.3)	1.4 (1.1; 1.9)	0.551
EPA (C20:5n3) (g)	0.13 (0.09, 0.23)	0.08 (0.04, 0.21)	0.165
Docosapentaenoic acid (C22:5n3) (g)	0.03 (0.02, 0.08)	0.04 (0.02, 0.06)	0.823
DHA (C22:6n3) (g)	0.45 (0.33, 0.56)	0.30 (0.17, 0.51)	**0.020**
EPA + DHA (g)	0.58 (0.47, 0.82	0.38 (0.22, 0.73)	**0.035**
Trans fatty acids (g)	0.46 (0.40, 0.61)	0.44 (0.29, 0.53)	0.170
Cholesterol (g)	343.6 (294.2, 438.2)	309.6 (266.2, 381.1)	0.185
Cholesterol (mg/1000 Kcal)	147.2 (135.3, 189.1)	138.9 (121.6, 165.8)	0.083
Thiamine (B1) (mg)	1.9 (1.3, 2.8)	1.9 (1.5, 2.4)	0.765
Riboflavin (B2) (mg)	2.6 (2.3, 3.6)	2.5 (1.8, 3.2)	0.298
Niacin (B3) (mg)	47.3 ()30.9, 60.9)	41.1 (34.8, 49.1)	0.536
Pantothenic acid (B5) (mg)	8.6 (5.4, 11.4)	7.1 (5.6, 10.2)	0.811
Pyridoxine (B6) (mg)	2.7 (2.2, 3.6)	3.7 (2.7, 4.3)	0.954
Biotin (B8) (μg)	43.7 (31.5, 77.7)	46.1 (31.3, 73.0)	0.913
Folate food + folic acid (B9) (μg)	478.4 (278.6, 678.2)	434,8 (320.3,617.8)	0.973
Cobalamin (B12) (μg)	7.5 (6.8, 8.5)	6.2 (5.1, 8.6)	**0.046**
Vitamin A (μg)	1441.2 (1107.4, 1981.2)	1243.2 (943.6, 1798.8)	0.216
Vitamin C (mg)	141.2 (89.2, 252.2)	165.3 (118.2, 234.6)	0.462
Vitamin D (μg)	6.8 (3.5, 7.8)	4.8 (2.3, 7.4)	0.197
Vitamin E (μg)	17.5 (11.8, 24.4)	15.7 (11.7, 22.2)	0.661
Calcium (mg)	1152.4 (918.0, 1429.2)	1111.5 (876.3, 1312.5)	0.737
Iodine (μg)	259.4 (141.6, 334.6)	236.6 (148.5, 331.2)	0.889
Iron (mg)	22.3 (12.5, 32.7)	19.3 (14.8, 28.5)	0.490
Phosphorus (mg)	1675.8 (1388.0, 1947.4)	1655.7 (1370.0, 1871.0)	0.784
Selenium (μg)	128.8 (98.9, 139.4)	112.7 (95.0, 135.1)	0.653
Zinc (mg)	13.4 (9.5, 20.7)	13.0 (10.3, 18.5)	0.998

Normally distributed continuous variable: mean (standard deviation).

Non-normally distributed continuous variable: median (25th, 75th percentile).

Values in bold: $p < 0.05$.

Abbreviations: DHA – docosahexaenoic acid, EPA – eicosapentaenoic acid, Kcal – kilocalories, MUFAs – monounsaturated fatty acids, PUFAs – polyunsaturated fatty acids, SFA – saturated fatty acids.

Table S5. The Prevalence of inadequate nutrient intake, regarding Harmonized Average Requirement (H-AR) proposed by Allen et al., recommended daily intakes from Ortega et al. and Acceptable Macronutrient Distribution Ranges from the Institute of Medicine according to study group: Mothers of hospitalized very-preterm infants (MHVPI) (**n**=15) and human milk donors (HMD) (**n**=110).

	**Cutoffs**[1]	**MHVPI**	**HMD**	**p-value**
			**n (%)**	
Energy (Kcal)	2430	10 (66.7)	71 (64.5)	1.000
Protein (g)	66	0.0 (0.0)	6 (5.5)	1.000
Kcal from carbohydrate (%)	45-65	11 (73.3) [2]	82 (74.5) [2]	0.519
Kcal from protein (%)	10-35	0.0 (0.0)	0.0 (0.0)	-
Kcal from fat (%)	20-35	13 (86.7) [3]	61 (55.5) [3]	0.267
Kcal from n-3 fatty acids (%)	0.6-1.2	100 (100) [3]	19 (17.3); [2]<br>11 (10.0) [3]	0.054
Thiamine (B1) (mg)	1.2	3 (20.0)	7 (6.4)	0.100
Riboflavin (B2) (mg)	1.7	2 (13.3)	18 (16.4)	1.000
Niacin (B3) (mg)	13	0 (0.0)	0 (0.0)	-
Pantothenic acid (B5) (mg)	5.6	5 (33.3)	27 (24.5)	0.530
Pyridoxine (B6) (mg)	1.4	1 (6.7)	2 (1.8)	0.321
Biotin (B8) (μg)	36	6 (40.0)	39 (35.5)	0.778
Folate food + folic acid (B9) (μg)	380 (DFE)	6 (40.0)	43 (39.1)	1.000
Cobalamin (B12) (μg)	2.4	0 (0.0)	0 (0.0)	-
Vitamin A (μg)	1020	2 (13.3)	37 (33.6)	0.143
Vitamin C (mg)	145	8 (53.3)	39 (35.5)	0.255
Vitamin D (μg)	10	13 (86.7)	96 (87.3)	1.000
Vitamin E (μg)	16	7 (46.7)	57 (51.8)	0.787
Calcium (mg)	750/860 [4]	3 (20.0)	11 (10.0)	0.374
Iodine (μg)	209	6 (40.0)	46 (41.8)	1.000
Iron (mg)	11.2 [5]	1 (6.7)	4 (3.6)	0.478
Phosphorus (mg)	580	0 (0.0)	0 (0.0)	-
Selenium (μg)	59	1 (6.7)	2 (1.8)	0.321
Zinc (mg)	10 [6]	5 (33.3)	23 (20.9)	0.323

H-AR – the harmonized average requirement, was proposed by Allen et al., after they selected values from the standards set by EFSA (for Europe) and the IOM (for the United States and Canada), giving priority to those published most recently.

[1] Cut-offs compiled by different sources including H-AR, Acceptable Macronutrient Distribution Ranges (Institute of Medicine) and Ortega et al.

[2] Below the inferior limit of the reference range.

[3] Above the superior limit of the reference range.

[4] In the case of calcium, the H-AR proposed for lactating mothers is 860 mg if they are ≤18-30 years and 750 mg if 31-50 years. We calculated inadequate nutrient intake corresponding to the age of each woman.

[5] In the case of iron, the H-AR values proposed depend on absorption (high, moderate, and low). Low absorption is characteristic of a plant-based diet. Because none of the investigated MHVPI followed a plant-based diet, therefore, we assumed a moderate absorption.

[6] In the case of zinc, the H-AR values proposed depend on phytate intake (refined, semi-refined, semi-unrefined, and unrefined). Due to the abandonment of the traditional mediterranean dietary patterns during the last decades, we assumed a semi-refined diet.

Values in bold: $p < 0.05$.

Table S6. Daily number of food servings, and intake of supplements and iodized salt determined using the 5-day dietary record according to study group: Mothers of hospitalized very-preterm infants (MHVPI) (*n*=15) and human milk donors (HMD) (*n*=110).

	**MHVPI**	**HMD**	**p-value**	**Recommendations [1]**
Servings by day:				
Dairy	2.6 (1.8, 3.1)	2.4 (1.6, 3.5)	0.927	≥ 4
Grains, legumes, and nuts	5.1 (1.8)	6.0 (1.9)	0.088	≥7
Vegetables and greens	3.2 (2.1, 3.8)	3.2 (2.4; 4.2)	0.335	≥4
Fruits	1.9 (0.8, 2.7)	1.8 (1.0, 2.5)	0.963	≥3
Eggs, Meat, and Fish	3.1 (1.0)	2.8 (1.1)	0.312	2-3
HEI	61.9 (8.3)	63.6 (8.9)	0.475	
Supplement intake (yes)	9 (64.3)	62 (56.4)	0.775	
Iodized salt intake (yes)	10 (66.7)	87 (82.1)	0.175	
Salt (g/day)	1.0 (0.6)	1.1 (0.6)	0.520	

Normally distributed continuous variable: Mean (standard deviation).

Non-normally distributed continuous variable: Median (25th, 75th percentile).

Qualitative variable: absolute (n) and relative frequencies (%).

[1] Number of recommended daily servings of food for lactating women.

Values in bold: $p < 0.05$.

Abbreviations: HEI – Health Eating Index.

Table S7. Number of food portions determined using the food consumption frequency questionnaire according to study group: Mothers of hospitalized very-preterm infants (MHVPI) and human milk donors (HMD).

	n	**MHVPI**	*n*	**DHM**	*p-value*	**Standard portion size[1]**
Milk (servings/day)	15	1.5 (0.6, 2.2)	110	1.3 (0.9, 2.2)	0.902	200-250 mL<br>Yogurt 200-250 g
Other dairy products (servings/day)	15	2.3 (0.8, 3.9)	107	1.5 (0.6, 2.3)	**0.039**	Fresh cheese 80-125 g,<br>Cured cheese 40-60 g
Meats and derivatives (servings/day)	15	0.9 (0.4, 1.3)	107	0.6 (0.4, 0.9)	0.242	100-125g
Fish (servings/week)	15	2.2 (1.1, 2.9)	106	1.9 (0.9-2.9)	0.995	125-150g
Eggs (servings/week)	15	2.8 (2.0, 3.5)	106	2.6 (1.7, 3.5)	0.871	60g
Fruits (servings/day)	15	1.3 (0.6, 3.1)	105	1.9 (1.0, 3.8)	0.341	120-200g
Raw vegetables (servings/day)	15	0.5 (0.2, 0.9)	106	0.5 (0.2, 0.9)	0.936	150-200g
Cooked vegetables (servings/day)	15	0.9 (0.6, 0.9)	105	0.7 (0.4, 1.2)	0.895	150-200g
Legumes (servings/week)	15	2.9 (1.4, 4.3)	107	2.9 (1.8, 4.3)	0.854	60-80g
Bread (servings/day)	15	1.0 (0.4, 1.6)	106	1.2 (0.7, 2.4)	0.362	40-60g
Pasta, rice, other grains (servings/week)	15	5.4 (3.6, 11.3)	106	5.7 (2.9, 9.9)	0.859	60-80g
Nuts (servings/week)	7	7.0 (0.4, 14.0)	25	3.5 (1.0, 6.9)	0.679	25g
Oils and fats (servings/day)	14	2.0 (0.9, 2.6)	106	2.0 (1.5, 3.0)	0.156	10g
Sweets (grams/week)	15	3.9 (0.8, 5.6)	106	3.9 (1.3, 6.1)	0.793	ALAP

Non-normally distributed continuous variable: median (25th, 75th percentile).

[1] Standard portion size as suggested by the Spanish Society of Community Nutrition. If a range was proposed, the mean of the two ranges were calculated [111].

Values in bold: $p < 0.05$.

Abbreviations: ALAP – as low as possible.

Table S8. Fatty acid composition (g/100 g of total fat) in the erythrocytes and plasma samples according to study group: Mothers of hospitalized very-preterm infants (MHVPI) (*n*=14) and human milk donors (HMD) (*n*=110).

Fatty Acid (%)	Common Name	MHVPI	HMD	p-value
	**Erythrocytes**			
**SFAs**				
C14:0	Myristic	0.12 (0.04)	0.12 (0.05)	0.824
DMA C16:0		1.96 (0.20)	2.19 (0.24)	**0.001**
C16:0	Palmitic	21.77 (1.44)	21.35 (2.04)	0.462
DMA C18:0		3.01 (0.25)	3.43 (0.34)	**0.000**
C18:0	Stearic	19.57 (18.01, 21.08)	20.25 (19.12, 21.08)	0.107
C24:0	Lignoceric	3.14 (2.19, 3.95)	2.30 (1.70, 2.84)	**0.011**
**MUFAs**				
C17:1	Margaroleic	0.34 (0.26, 0.38)	0.32 (0.26, 0.41)	0.599
C18:1 *cis*-11 (n7)	*Cis* vaccenic	0.27 (0.24, 0.32)	0.23 (0.19, 0.28)	**0.010**
C18:1 *cis*-9 (n9)	Oleic	11.86 (1.80)	12.59 (1.44)	0.085
**n-6 PUFAs**				
C18:2 (n6)	Linoleic	9.31 (1.74)	8.15 (1.40)	**0.006**
C20:3 (n6)	Dihomo-γ -linolenic	1.51 (1.24, 2.29)	0.95 (0.67, 1.21)	**0.000**
C20:4 (n6)	Arachidonic	22.14 (4.25)	24.26 (3.00)	**0.002**
**n-3 PUFAs**				
C20:5 (n3)	Eicosapentaenoic	0.00 (0.00, 0.31)	0.00 (0.00, 0.23)	0.830
C22:5 (n3)	Docosapentaenoic	0.83 (0.25)	0.71 (0.33)	0.207
C22:6 (n3)	Docosahexaenoic	3.97 (1.23)	2.91 (1.20)	**0.002**
**Fatty Acid Families**				
DMAs		4.97 (0.36)	5.62 (0.49)	**0.000**
SFAs		44.43 (2.52)	44.06 (2.67)	0.621
MUFAs		12.49 (1.90)	13.17 (1.48)	0.122
PUFAs		36.86 (35.62, 42.09)	37.26 (34.94, 39.01)	0.437
MCFAs		0.12 (0.04)	0.12 (0.05)	0.824
LCFAs		62.89 (4.74)	62.90 (4.36)	0.993
VLCFAs		28.81 (4.04)	29.01 (3.68)	0.848
n-6 PUFAs		33.20 (3.36)	33.42 (2.86)	0.793
n-3 PUFAs		4.92 (1.47)	3.74 (1.59)	**0.010**
n-6 PUFAs/n-3 PUFAs		5.70 (5.37, 9.43)	9.53 (7.16, 12.37)	**0.044**
	**Plasma[1]**			
**SFAs**				
C14:0	Myristic	0.41 (0.19, 0.49)	0.27 (0.20, 0.32)	0.059
C15:0	Pentadecylic	0.05 (0.04, 0.06)	0.04 (0.03, 0.06)	0.135
DMA C16:0		0.16 (0.12, 0.19)	0.19 (0.15, 0.24)	**0.038**
C16:0	Palmitic	20.41 (19.26, 21.28)	21.25 (20.11, 22.27)	0.087
DMA C18:0		0.07 (0.05, 0.09)	0.07 (0.05, 0.11)	0.487
C18:0	Stearic	6.21 (5.76, 6.61)	6.19 (5.80, 6.70)	0.784
**MUFAs**				
C16:1 *cis*-9 (n7)	Palmitoleic	0.55 (0.43, 0.77)	0.42 (0.30, 0.54)	**0.004**
C18:1 *cis*-11 (n7)	*Cis* vaccenic	0.52 (0.44, 0.66)	0.41 (0.32, 0.54)	**0.011**
C18:1 *cis*-9 (n9)	Oleic	18.13 (16.21, 21.42)	17.98 (16.53, 19.78)	1.000
**n-6 PUFAs**				
C18:2 (n6)	Linoleic	40.56 (35.27, 44.46)	39.33 (35.88, 42.46)	0.563
C20:3 (n6)	Dihomo-γ -linolenic	1.39 (1.19, 1.82)	1.03 (0.68, 1.48)	**0.013**
C20:4 (n6)	Arachidonic	10.42 (2.96)	11.26 (3.15)	0.346
**n-3 PUFAs**				
C20:5 (n3)	Eicosapentaenoic	0.17 (0.14, 0.36)	0.14 (0.00, 0.37)	0.208
C22:6 (n3)	Docosahexaenoic	0.98 (0.87, 1.52)	0.69 (0.46, 1.07)	**0.030**
**Fatty Acid Families**				
DMAs		0.22 (0.17, 0.29)	0.27 (0.20, 0.36)	0.097

Fatty Acid (%)	Common Name	MHVPI	HMD	p-value
SFAs		26.91 (25.76, 28.34)	27.86 (26.57, 29.07)	0.134
MUFAs		19.07 (17.11, 22.74	18.94 (17.43, 20.84)	0.787
PUFAs		54.77 (48.89, 56.92)	53.01 (50.52, 55.19)	0.357
MCFAs		0.46 (0.26, 0.55)	0.30 (0.25, 0.38)	**0.044**
LCFAs		86.10 (3.09)	85.90 (3.94)	0.858
VLCFAs		12.90 (10.10, 15.77)	13.11 (10.95, 16.32)	0.898
n-6 PUFAs		53.11 (48.01, 55.47)	51.79 (49.41, 54.13)	0.561
n-3 PUFAs		1.26 (1.03, 1.70)	0.85 (0.47, 1.41)	**0.047**
n-6 PUFAs/n-3 PUFAs		40.16 (32.92, 51.41)	64.43 (35.47, 111.96)	0.066

Normally distributed continuous variable: mean (standard deviation).

No normally distributed continuous variable: median (25th, 75th percentile).

[1] HMD: *n*=109 , MHVPI: *n*=14.

Values in bold: $p < 0.05$.

Abbreviations: DMA – dimethylacetal, LCFAs – long-chain fatty acids, MCFAs – medium-chain fatty acids, MUFAs – monounsaturated fatty acids, PUFAs – poly unsaturated fatty acids, SCFAs – short-chain fatty acids, SFAs – saturated fatty acids and VLCFAs – very-long-chain fatty acids.

Table S9. Concentrations of nutrients and biochemical determinations in the erythrocytes, plasma, and urine according to study group: Mothers of hospitalized very-preterm infants (MHVPI) and human milk donors (HMD).

Variable[1]	Unit	*n*	MHVPI	*n*	HMD	p-value
			**Erythrocytes**			
**Haemoglobin**	g/dL	15	25.13 (21.60, 26.23)	110	25.58 (23.80, 27.67	0.121
**EGRAC**		9	1.09 (0.17)	90	1.24 (0.24)	0.080
**Riboflavin, B2**	ng/L	15	948,00 (499.30, 1195.80)	110	745.75 (548.30, 958.70)	0.481
	ng/hHb		3.51 (1.99, 5.00)		2.91 (1.96, 4.01)	0.351
**Nicotinamide, B3**	μg/L	15	7572.00 (6411.10, 8684.30)	110	5089.40 (1138.02, 6595.35)	**<0.001**
	μg/g Hb		32.27 (28.12, 37.24)		18.55 (5.00, 26.24)	**<0.001**
**Pantothenic acid, B5**	μg/L	15	12.20 (6.10, 16.20)	110	29.20 (17.02, 65.25)	**<0.001**
	mg/g Hb		48.22 (26.87, 73.60)		114.09 (65.51, 231.25)	**<0.001**
**Pyridoxamine, B6**	μg/L	15	497.00 (454.70, 606.70)	110	533.35 (411.47, 668.38)	0.926
	μg/g Hb		2.14 (1.83, 2.87)		1.97 (1.52, 2.71)	0.466
			**Plasma**			
**Thiamin, B1**	μg/L	15	0.32 (0.14, 1.08)	110	0.31 (0.19, 0.56)	0.689
**Riboflavin, B2**	μg/L	15	19.35 (15.46, 34.70)	110	16.79 (12.90, 24.98)	0.375
**Nicotinamide, B3**	μg/L	15	4.82 (2.87, 5.80)	110	3.87 (3.05, 5.64)	0.387
**Pantothenic acid, B5**	μg/L	15	132.98 (88.60, 159.31)	110	122.75 (96.41, 154.21)	0.773
**Pyridoxine, B6**	μg/L	15	151.49 (120.78, 162.17)	110	133.55 (115.33, 161.84)	0.211
**Pyridoxamine, B6**	μg/L	15	258.16 (234.05, 280.06)	110	262.18 (242.84, 284.85)	0.444
**Folic acid, B9**	μg/L	15	2.36 (1.77, 3.27)	110	2.39 (1.55, 3.31)	0.945
**Cobalamin, B12**	pM	15	409.00 (342.00, 524.00)	110	510.50 (404.50, 647.50)	**0.019**
**Holotranscobalamin II**	pM	15	176.50 (131.00, 214.00)	110	176.35 (135.45, 223.38)	0.441
**Homocysteine**	μg/M	15	11.41 (3.73)	110	10.27 (3.48)	0.239
**Retinol, A**	μg/dL	15	60.76 (11.88)	109	50.33 (16.87)	**0.022**
**Ascorbic acid, C**	μM	15	65.90 (44.50, 92.90)	110	44.70 (29.98, 64.38)	**0.034**
**$25(OH)D_3$**	ng/mL	15	7.06 (4.78, 8.87)	110	6.60 (3.82, 9.63)	0.891
**$1,25(OH)D_2$**	pg/mL	15	94.40 (71.60, 318.41)	110	98.32 (64.59, 144.04)	0.279
**α-tocopherol, E**	μg/dL	15	248.90 (187.70, 914.10)	108	223.35 (167.00, 293.40)	0.220
**α -tocopherol: total lipids** (cholesterol+triacylglycerols)	μmol/μmol					
**α -tocopherol: cholesterol**	μmol/μmol	15				
**γ-tocopherol, E**	μg/dL	15	50.50 (42.50, 68.20)	105	35.40 (21.25, 48.70)	**0.002**
**Total cholesterol**	mg/dL	15	219.50 (183.20, 257.80)	110	185.80 (162.80, 200.98)	**0.004**
**Triacylglycerols**	mg/dL	15	57.40 (46.70, 92.30)	110	43.70 (36.95, 56.05)	**0.002**
**HDL**	mg/dL	15	63.20 (59.10, 67.50)	110	61.10 (55.80, 66.83)	0.313
**LDL**	mg/dL	15	118.30 (109.40, 154.30)	110	102.45 (86.25, 117.23)	**0.008**
			**Urine**			
**Cr**	mg/dL		90.78 (44.64)	110	122.68 (50.91)	**0.023**
**Methylmalonic acid**	mg/L	15	4.59 (2.77, 7.52)	109	6.30 (3.99, 7.65)	0.260
	μg/mg Cr	15	4.88 (3.76, 8.61)		4.83 (3.46, 6.79)	0.472
**Calcium**	mg/L	15	91.90 (37.00, 151.00)	110	77.51 (44.95, 143.23)	0.888
	mg/mg Cr	15	0076 (0.59, 0.84)	110	0.84 (0.63, 1.06)	0.095
**Iodine**	μg/L	15	106.04 (67.93, 141.60)	110	116.66 (76.90, 160.72)	0.388
	μg/mg Cr	15	0.14 (0.10, 0.17)	110	0.10 (0.07, 0.15)	0.112
**Phosphorus**	mg/L	15	717,70 (322.40, 875.44)	110	961.74 (696.50, 1423.85)	**0.004**
	mg/mg Cr	15	0.11 (0.07, 0.18)	110	0.07 (0.04, 0.12)	0.056
**Sodium**	mg/L	15	2898.89 (1450.41)	110	3376.72 (1335.01)	0.200
	mg/mg Cr	15	3.51 (2.07,5.18)	110	3.00 (2.04, 3.74)	0.226

Normally distributed continuous variable: mean (standard deviation).

Non-normally distributed continuous variable: median (25th, 75th percentile).

[1] The units of our results have been converted to the international system.

Values in bold: $p < 0.05$.

Abbreviations: Cr – creatinine, EGRAC – erythrocyte glutathione reductase activity coefficient, M – molar, *n* – number of samples, SE – standard error.

Table S10. Lipid classes profile, molecular species of triacylglycerol content regarding their carbon number (CN), and relative composition of phospholipids in human milk according to study group: Mothers of hospitalized very-preterm infants (MHVPI) (*n*=12) and human milk donors (HMD) (*n*=20).

	**MHVPI**	**Donors**	**p-value**
	**Lipid classes**	**(g/100g fat)**	
Triacylglycerols	96.85 (2.15)	95.46 (2.37)	0.105
Diacylglycerols	2.77 (1.98)	4.07 (2.14)	0.097
Monoacylglycerols	0.02 (0.01, 0.04)	0.03 (0.02, 0.07)	0.072
Free fatty acids + cholesterol	0.31 (0.13, 0.39)	0.31 (0.22, 0.51)	0.267
Polar lipids	0.05 (0.05, 0.07)	0.05 (0.04, 0.06)	0.253
	**Triacylglycerols**	**(g/100g fat)**	
CN24	0.01 (0.01, 0.01)	0.01 (0.01, 0.02)	0.066
CN26	0.08 (0.07, 0.12)	0.10 (0.09, 0.11)	0.157
CN28	0.04 (0.01, 0.05)	0.07 (0.05, 0.12)	**0.002**
CN30	0.09 (0.03, 0.11)	0.19 (0.13, 0.28)	**<0.001**
CN32	0.09 (0.06, 0.17)	0.26 (0.18, 0.42)	**<0.001**
CN34	0.18 (0.06, 0.27)	0.33 (0.12, 0.46)	0.065
CN36	0.50 (0.17)	0.43 (0.28)	0.448
CN38	1.28 (0.34)	1.57 (0.68)	0.178
CN40	2.07 (0.64)	2.02 (0.54)	0.848
CN42	3.08 (1.48)	2.70 (0.91)	0.436
CN44	5.47 (2.05)	5.02 (1.37)	0.464
CN46	7.85 (2.44)	7.51 (1.50)	0.670
CN48	10.65 (2.42)	10.72 (1.43)	0.929
CN50	14.47 (1.90)	14.71 (2.16)	0.756
CN52	36.76 (6.25)	36.89 (4.83)	0.949
CN54	15.97 (13.21, 20.12)	17.38 (13.25, 18.97)	0.744
	**Phospholipids**	**(g/100g polar lipids)**	
Phosphatidylethanolamine	34.18 (8.33)	24.63 (7.88)	**0.003**
Phosphatidylcholine	36.77 (6.46)	30.95 (5.00)	**0.008**
Sphingomyelin	29.05 (8.99)	44.43 (11.09)	**<0.001**

Normally distributed continuous variable: mean (standard deviation).

Non-normally distributed continuous variable: median (25th, 75th percentile).

Values in bold: $p < 0.05$.

Table S11. Fatty acid composition (g/100 g of total fat) in human milk according to study group: Mothers of hospitalized very-preterm infants (MHVPI) (*n*=14) and human milk donors (HMD) (*n*=106).

Fatty Acid (%)	Common Name	MHVPI	Donors	p-value
		**SFAs**		
C6:0	Caproic	0.09 (0.08, 0.09)	0.11 (0.10, 0.12)	**< 0.001**
C8:0	Caprylic	0.21 (0.15, 0.24)	0.18 (0.16, 0.21)	0.338
C10:0	Capric	1.27 (0.35)	1.19 (0.28)	0.337
C12:0	Lauric	5.15 (1.82)	5.42 (1.59)	0.557
C14:0	Myristic	5.38 (3.54, 7.55)	5.89 (4.89, 7.89)	0.136
C15:0		0.17 (0.13, 0.23)	0.18 (0.13, 0.25)	0.580
C15:0 ai	C15:0 anteiso	0.02 (0.02, 0.03)	0.02 (0.02, 0.03)	0.515
C15:0 i	C15:0 iso	0.03 (0.02, 0.05)	0.03 (0.02, 0.05)	0.552
C16:0	Palmitic	19.64 (2.13)	19.63 (2.46)	0.990
C16:0 i	C16:0 iso	0.02 (0.01, 0.02)	0.02 (0.01, 0.03)	0.323
C17:0	Margaric	0.18 (0.15, 0.21)	0.19 (0.15, 0.23)	0.469
C17:0 ai	C17:0 anteiso	0.04 (0.03, 0.05)	0.04 (0.03, 0.06)	0.444
C17:0 i	C17:0 iso	0.27 (0.08)	0.27 (0.06)	0.833
C18:0	Stearic	6.11 (1.01)	5.79 (1.25)	0.357
C20:0	Arachidic	0.17 (0.12, 0.24)	0.16 (0.11, 0.21)	0.452
		**MUFAs**		
C14:1 *cis*-9 (n5)	Myristoleic	0.06 (0.04, 0.11)	0.07 (0.04, 0.11)	0.672
C16:1 *cis*-9 (n7)	Palmitoleic	1.47 (1.10, 1.66)	1.47 (1.19, 1.77)	0.313
C17:1	Margaroleic	0.06 (0.03)	0.07 (0.03)	0.154
∑ C18:1 *trans*		0.16 (0.10. 0.24)	0.22 (0.12, 0.34)	0.207
C18:1 *cis*-9 (n9)	Oleic	37.33 (6.09)	38.30 (4.99)	0.506
C18:1 *cis*-11 (n7)	*Cis* vaccenic	1.49 (0.24)	1.61 (0.28)	0.140
C20:1 (n9)	Gondoic	0.78 (0.35, 0.97)	0.53 (0.37, 0.83)	0.396
	**n-6**	**PUFAs**		
C18:2 (n6)	Linoleic	16.90 (13.97, 18.77)	14.69 (12.28, 17.17)	0.062
C20:2 (n6)	Eicosadienoic	0.39 (0.30, 0.55)	0.25 (0.19, 0.35)	**< 0.001**
C20:3 (n6)	Dihomo-γ -linolenic	0.45 (0.37, 0.60)	0.33 (0.23, 0.43)	**0.001**
C20:4 (n6)	Arachidonic	0.54 (0.20)	0.54 (0.17)	0.956
	**n-3**	**PUFAs**		
C18:3 (n3)	Linolenic (ALA)	0.67 (0.52, 0.84)	0.50 (0.40, 0.61)	**0.006**
C22:5 (n3)	Docosapentaenoic	0.08 (0.04, 0.11)	0.07 (0.05, 0.11)	0.969
C22:6 (n3)	Docosahexaenoic	0.41 (0.17, 0.49)	0.27 (0.16, 0.45)	0.384
	**n-7**	**PUFAs**		
C18:2 c9, t11 (n7)	Rumenic	0.04 (0.03, 0.08)	0.08 (0.04, 0.12)	0.064
		**Fatty Acid**	**Families**	
Not identified		0.23 (0.15, 0.30)	0.20 (0.14, 0.25)	0.211
SFAs		39.06 (35.77, 42.42)	39.83 (36.92, 42.17)	0.606
MUFAs		41.31 (6.24)	42.57 (5.23)	0.411
PUFAs		19.55 (16.54, 21.69)	16.70 (14.70, 19.36)	**0.026**
SCFAs		0.09 (0.08, 0.09)	0.11 (0.10, 0.12)	**< 0.001**
MCFAs (C8-C15)		11.68 (8.75, 16.67)	13.02 (11.11, 16.30)	0.350
LCFAs (C16-C18)		84.32 (79.77, 88.19)	84.00 (80.56, 86.35)	0.676
VLCFAs (C20-C24)		2.94 (2.55, 3.55)	2.32 ()1.99, 3.09)	**0.012**
n-6 PUFAs		18.27 (15.34, 20.66)	15.75 (13.44, 18.42)	**0.037**
n-3 PUFAs		1.09 (0.82, 1.43)	0.86 (0.71, 1.15)	0.094
n-6 PUFAs/n-3 PUFAs		15.19 (11.76, 24.08)	17.28 (13.37, 24.93)	0.387
LA/ALA ratio		20.36 (18.22, 36.38)	29.02 (22.55, 40.48)	0.098
ARA/DHA ratio		1.31 (0.94, 3.35)	1.86 (1.33, 3.08)	0.241

Normally distributed continuous variable: mean (standard deviation).

Non-normally distributed continuous variable: median (25th, 75th percentile).

Mean standard derivation are shown after the semicolons for comparison with reference values.

Values in bold: $p < 0.05$.

Abbreviations: LCFAs – long-chain fatty acids, MCFAs – medium-chain fatty acids, MUFAs – monounsaturated fatty acids, PUFAs – poly unsaturated fatty acids, SCFAs – short-chain fatty acids, SFAs – saturated fatty acids and VLCFAs – very-long-chain fatty acids.

Table S12. Macronutrient, vitamin and mineral composition in the human milk according to study group: Mothers of hospitalized very-preterm infants (MHVPI) and human milk donors (HMD).

**Variable**[1]	**Unit**	***n***	**MHVPI**	***n***	**HMD**	**p-value**
			**Macronutrients**			
Lipids	g/dL	15	3.36 (2.27, 3.95)	100	2.87 (2.04, 4.37)	0.852
Carbohydrates	g/dL	15	7.75 (7.57, 7.96)	100	7.79 (7.58, 7.94)	0.910
Proteins	g/dL	15	1.36 (1.17, 1.45)	100	1.19 (1.04, 1.3)	**0.006**
			**Vitamins**			
Retinol, A	μg/dL	15	47.75 (36.65, 69.25)	109	40.83 (26.50, 73.69)	0.192
Free thiamin, B1	μg/L	15	10.70 (7.53, 22.13)	110	18.00 (9.89, 28.03)	0.091
Free riboflavin, B2	μg/L	15	32.98 (12.33, 71.70)	110	47.71 (23.11, 100.53)	0.187
Nicotinamide, B3	μg/L	15	55.73 (35.78. 95.03)	110	45.99 (27.12, 81.41)	0.444
Pantothenic acid, B5	μg/L	15	2334.63 (1795.30, 2634.95)	110	2248.13 (1800.11, 2535.66)	0.848
Pyridoxal, B6	μg/L	15	24.43 (18.98, 68.93)	110	36.35 (26.83, 53.19)	0.787
Folic acid, B9	μg/L	15	19.88 (7.04)	110	19.80 (7.05)	0.970
Cobalamin, B12	pM/ **pmol/L**	15	478.08 (456.95, 561.93)	109	482.30 (445.25, 530.89)	0.728
Ascorbic acid, C	μg/L **mg/dL**	15	4.51 (2.75)	109	3.91 (1.73)	0.250
Dehydroascorbic acid, C	μg/L **mg/dL**	15	1.98 (1.07, 2.57)	109	1.91 (1.32, 3.45)	0.512
Vitamin C total *	μg/dL	15	6.27 (4.51, 7.29)	109	6.41 (5.50, 7.04)	0.728
Vitamin D_3, D	pg/mL	15	805.38 (151.17, 4142.30)	110	1614.21 (369.43, 5313.55)	0.269
25(OH)D_3, D	pg/mL	15	66.23 (35.05, 171.13)	110	53.05 (26.86, 110.66)	0.355
α-tocopherol, E	μg/dL	15	415.03 (383.00, 547.35)	109	457.15 (370.96, 591.81)	0.797
γ-tocopherol, E	μg/dL	15	40.48 (35.33, 55.85)	109	67.21 (50.45, 85.35)	0.187
			**Minerals**			
Calcium	μg/L	15	108.75 (63.40, 125.40)	110	98.98 (59.58, 127.78)	0.617
Iodine	mg/L	15	236.25 (137.08, 338.98)	110	148.77 (96.00, 205.30)	**0.021**
Phosphorous	mg/L	15	131.36 (23.45)	110	132.66 (27.25)	0.860
Selenium	μg/L	15	13.38 (10.65, 16.08)	110	10.99 (9.24, 12.93)	**0.004**

Normally distributed continuous variable: mean (standard deviation).

Non-normally distributed continuous variable: median (25th, 75th percentile).

For the purpose of comparison, the units of our results were converted to those used in the references for the nutrients in human milk.

* Vitamin C = ascorbic acid + dehydroascorbic acid.

Values in bold: $p < 0.05$.

Abbreviations: SE – standard error; IQR – interquartile range; TE – tocopherol equivalents.
